# Proteobacteria explain significant functional variability in the human gut microbiome

**DOI:** 10.1186/s40168-017-0244-z

**Published:** 2017-03-23

**Authors:** Patrick H. Bradley, Katherine S. Pollard

**Affiliations:** 1 Gladstone Institutes, San Francisco, CA USA; 2 Division of Biostatistics, Institute for Human Genetics, and Institute for Computational Health Sciences, University of California, San Francisco, CA USA

**Keywords:** Human gut microbiome, Proteobacteria, Bacteroidetes, Firmicutes, Variance, Shotgun metagenomics, Statistical methods, Functional redundancy, Enterotypes, Human gut microbiome

## Abstract

**Background:**

While human gut microbiomes vary significantly in taxonomic composition, biological pathway abundance is surprisingly invariable across hosts. We hypothesized that healthy microbiomes appear functionally redundant due to factors that obscure differences in gene abundance between individuals.

**Results:**

To account for these biases, we developed a powerful test of gene variability called CCoDA, which is applicable to shotgun metagenomes from any environment and can integrate data from multiple studies. Our analysis of healthy human fecal metagenomes from three separate cohorts revealed thousands of genes whose abundance differs significantly and consistently between people, including glycolytic enzymes, lipopolysaccharide biosynthetic genes, and secretion systems. Even housekeeping pathways contain a mix of variable and invariable genes, though most highly conserved genes are significantly invariable. Variable genes tend to be associated with Proteobacteria, as opposed to taxa used to define enterotypes or the dominant phyla Bacteroidetes and Firmicutes.

**Conclusions:**

These results establish limits on functional redundancy and predict specific genes and taxa that may explain physiological differences between gut microbiomes.

**Electronic supplementary material:**

The online version of this article (doi:10.1186/s40168-017-0244-z) contains supplementary material, which is available to authorized users.

## Background

The microbes that inhabit the mammalian gut encode a wealth of proteins that contribute to a broad range of biological functions, from modulating the immune system [1–3] to participating in metabolism [4, 5]. Shotgun metagenomics is revolutionizing our ability to identify protein-coding genes from these microbes and associate gene levels with disease [6], drug efficacy [7] or side-effects [8], and other host traits. For instance, human gut microbiota associated with a traditional high-fiber agrarian diet encoded gene families involved in cellulose and xylan hydrolysis, which were absent in age-matched controls eating a typical Western diet [9]. The functional capabilities of the human gut microbiome go beyond statistical associations. A number of microbial genes have now been causally linked to host physiology. Examples include the colitis-inducing cytolethal distending toxins of *Helicobacter hepaticus* [10] and the enzymes of commensal bacteria that protect against these toxins by producing anti-inflammatory polysaccharide A [11].

It is therefore surprising that healthy human gut microbiomes have been characterized as functionally stable, with largely redundant gene repertoires in different hosts. We refer to these metagenomic gene families with very low variance in abundance across hosts as “invariable.” Several lines of evidence support this conclusion. First, biological pathway abundance tends to be less variable across metagenomes than it is between isolate genomes [12], suggesting strong selection for microbes that encode functions necessary for adaptation to the gut environment. Second, the relative abundances of pathways are strikingly invariable compared to the relative abundances of bacterial phyla in the same metagenomes [12, 13]. Thus, it appears that humans harbor phylogenetically distinct gut communities that all do more or less the same things, except in the context of disease or other extreme host phenotypes.

Functional redundancy deserves a closer look, however, because physiologically meaningful differences in gene abundances between healthy human microbiomes could easily have been missed. One primary factor may be that prior work did not look at quantitative abundances of individual genes but instead mainly summarized function at the level of Clusters of Orthologous Groups (COG) categories (e.g., “carbohydrate metabolism and transport”) and KEGG modules (e.g., “citrate cycle”) [12–14]. This strategy lacks the power to detect one component of a pathway or protein complex that varies in abundance across hosts if other components are less variable. This masking of variable genes (i.e., genes with high variance) is likely because the presence and abundance of most COG categories and KEGG modules will be dominated by core components (i.e., housekeeping genes) that are widely distributed across the tree of life and abundant in metagenomes. The only previous analyses of individual genes asked whether they were universally detected across all individuals sampled [12, 14]. However, universally detected genes may still vary substantially in abundance, and conversely, lower-abundance invariable genes may not be universally detected merely due to sampling. This approach is also sensitive to read depth [12] and sample size [14]. Based on these observations, we were motivated to quantitatively investigate functional redundancy at the level of individual sets of orthologs (or “gene families”).

To enable high-resolution, quantitative analysis of functional stability in the microbiome, we developed a statistical test that identifies individual gene families whose abundances are either significantly variable or invariable across samples. We named this test CCoDA, for Covariate-Corrected Dispersion Analysis. The inputs to the method are gene abundance values (e.g., normalized counts of metagenomic reads mapping to a particular gene), and the outputs are lists of genes whose abundances differ significantly more or less than expected across samples, which can be summarized by pathways and by the taxonomic groups contributing reads.

The study of variability, in addition to the more common study of average abundances, is becoming more popular in other areas of genomics, such as gene expression across tissues [15], epigenetic variation [16], and, especially, individual cells [17–21]. However, there are still few existing statistical approaches for determining whether a given observed amount of biological variability exceeds or falls beneath expectations, and the existing methods require the use of spike-ins to decompose technical and biological variability [19, 20]. Our method does not require these additional data, which are often not available in existing studies of the microbiome. Additionally, it incorporates solutions to three major challenges to studying functional redundancy with shotgun metagenomics data.

The first key innovation of our approach is using a test statistic that captures residual variability after accounting for the overall gene abundance. Like modern approaches for RNAseq analysis [22, 23] and proteomics analysis [24], we use the negative binomial distribution to directly model the sequencing count data and account for the mean-variance relationship. However, instead of using this distribution to more accurately detect genes with differences in abundance between groups, we use it to discover genes whose variances are unexpected given their mean values. This modeling choice is important because abundant genes will be variable just by chance due to the correlation between mean and variance in any sequencing experiment. Conversely, phylogenetically restricted genes will have relatively low variance due to being less abundant. Furthermore, gene abundances can be sparse (i.e., zero in many samples). For all of these reasons, simply ranking genes based on their variances would yield many false positives and false negatives.

A second benefit of our modeling approach is that we can adjust for systematic differences in a gene’s measured level between studies to allow for quantitative integration of data from multiple sources. Meta-analysis is essential for gaining sufficient power to detect variable genes across the range of mean abundance levels. It also ensures robustness and generalizability of discovered inter-individual differences, which occur by chance in small sets of metagenomes. Our modeling approach is also flexible enough to account for factors such as average genome size that can affect measurements of gene abundances.

Finally, our method does not require predefined cases and controls but instead enables discovery of genes that explain functional differences between microbiomes without prior knowledge of which groups of samples to compare. This is critical for the current phase of microbiome research, when many factors influencing microbial community composition are unknown. Gene families that contribute to survival in one particular type of healthy gut environment should emerge as variable between hosts and their functions may point to factors influencing community composition, mechanisms of microbe-host interactions, and biomarkers of presymptomic disease (e.g., pre-diabetes).

We applied CCoDA to healthy gut metagenomes (*n*=123) spanning three different shotgun sequencing studies and found both significantly invariable (3768) and variable (1219) gene families (false discovery rate (FDR) <5%). Many pathways, including some commonly viewed as housekeeping or previously identified as invariable across gut microbiota (e.g., central carbon metabolism and secretion), included significantly variable gene families. Phylogenetic distribution (PD) correlated overall with variability in gene family abundance, and exceptions to this trend highlight functions that may be involved in adaptation, such as two-component signaling and specialized secretion systems. This approach to discovering functions that distinguish microbial communities is applicable to any body site or environment.

Finally, the human gut is normally dominated by the bacterial phyla Bacteroidetes and Firmicutes [13]. Clades within these phyla (especially *Bacteroides*, *Prevotella*, and *Ruminococcaceae*) are the most commonly used to cluster individuals together into “enterotypes” [25–28] because they explain the most taxonomic variation. The Bacteroidetes-to-Firmicutes ratio has also been measured as a potential biomarker of interest in its own right [29–31]. In contrast, we show that the less abundant phylum Proteobacteria, and not Bacteroidetes or Firmicutes, is a major source of genes with the greatest variability in abundance across hosts. Thus, while Bacteroidetes and Firmicutes may contribute most to taxonomic variation between hosts, the abundance of Proteobacteria may capture more of the functional variation. This has implications for the interpretation of taxonomic data from human gut microbiota and, because of the link between Proteobacteria and dysbiosis [32], also suggests a potential relationship between inflammation and gene-level differences in gut microbial functions.

## Results

To describe variation within healthy gut microbiota across different human populations, we randomly selected 123 metagenomes of healthy individuals from the Human Microbiome Project (HMP, *n*=42) [13], controls in a study of type II diabetes (T2D, *n*=44) [33], and controls in a study of glucose control (GC, *n*=37) [34]. These span American, Chinese, and European populations, respectively (see the “Methods” section). We mapped these metagenomes to KEGG Orthology (KO) families with ShotMAP [35] and counted reads for 17,417 gene families.

Accurately normalizing gene read counts so that they are comparable across samples and studies is critical to our meta-analytical approach and any quantitative evaluation of shotgun metagenomes. We therefore quantified gene family abundance using reads per kilobase of genome equivalents (RPKG) [36]. This method of calculating abundances takes into account differences in the average genome size within different metagenomes, as well as factors such as gene length, that can also bias counts (long genes will generally have a greater proportion of reads).

### Unadjusted calculation of gene variability yields misleading results

One straight-forward approach to determining gene family variability, which has previously been employed in the literature [13], would simply be to calculate the variance of gene family abundances across all datasets. The tails of this distribution—for example, the top and bottom 10%—could then be termed “variable” and “invariable” gene families. However, by this metric, the most “variable” gene families would actually be enriched for pathways such as the ribosome (FDR-corrected *p* value *q*=2.4×10^−10^), DNA replication (*q*=0.07), and aminoacyl-transfer RNA (tRNA) biosynthesis (*q*=1.2×10^−6^). These results contradict biological intuition: it would be very surprising for genes within the best-conserved “housekeeping” pathways to be among the most variable, since they appear in most microbial genomes. (Here, we define “housekeeping” gene families as those involved in fundamental, highly conserved cellular processes such as translation, DNA replication, and central metabolism). Indeed, out of a recent list of 74 protein-coding genes that were universally present and single-copy in bacterial genomes, 14 were ribosomal genes and 10 were tRNA synthetases or tRNA modification enzymes [37]; “housekeeping” pathways also dominated previous lists of bacterial universal and single-copy genes [38].

Furthermore, according to this same straight-forward metric, the least variable gene families would include those involved in disease signatures such as “salmonella infection” (*q*=0.027), “pertussis” (*q*=1.4×10^−3^), and “legionellosis” (*q*=4.9×10^−3^). The presence of genes in these disease signatures does not necessarily indicate the presence of that disease or an active infection. However, it seems unlikely for genes involved in pathogenicity to be among the most stable components of the healthy human microbiome.

**Fig. 1:**
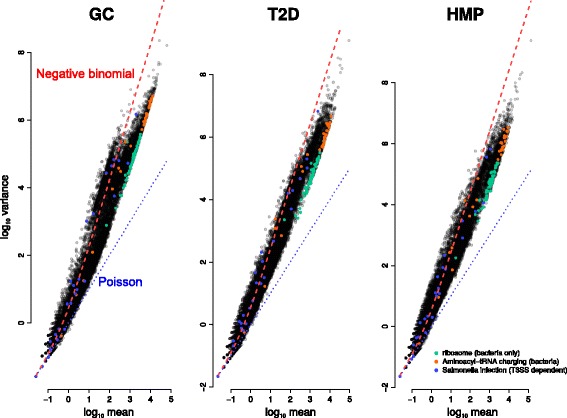
Shotgun metagenomic data show a very strong mean-variance relationship. The log10(mean) is plotted against log10(variance) for each gene family (points) in each study (headings). Bacterial ribosomal proteins (*green*), aminoacyl-tRNA charging genes (*orange*), and genes annotated to the T3SS-dependent *Salmonella* pathogenesis signature in KEGG (*blue*) are highlighted. *Trend lines* show a Poisson (*dashed blue line*) and a negative binomial (*dashed red line*) fit to the count data. Negative binomial provides a better fit in all three data sets

The explanation for this counterintuitive result can be visualized by plotting the mean vs. variance for each measured gene family (Fig. 1): in shotgun metagenomic data, mean and variance are tightly correlated over the entire range of means. This phenomenon is robust to the number of samples assessed (Additional file 1: Figure S1). Similar mean-variance relationships are actually observed in other high-throughput sequencing applications, such as RNAseq [39, 40] (which is why standard hypothesis tests based on assuming normality are inappropriate for RNAseq data, if the correct variance-stabilizing transformations are not applied [40]).

**Fig. 2:**
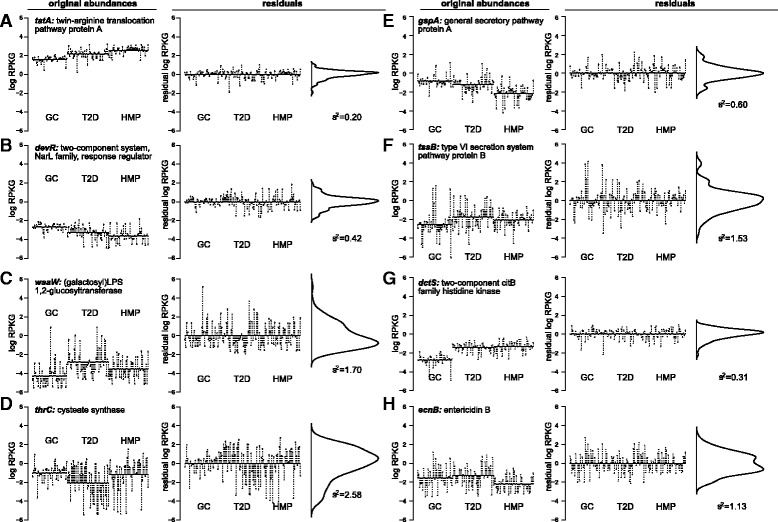
The residual variance statistic captures variation in gene families after accounting for between-study variation. The *left-hand panels* (“original abundances") show *filled circles* representing log-RPKG abundances for gene families from the KEGG Orthology (KO), with per-study means shown in *solid horizontal lines* and the distance from these means shown as *dashed vertical lines*. The *right-hand panels* (“residuals") show the same gene families after fitting a linear model that accounts for these per-study means, with an accompanying density plot showing the distribution of these residuals. $V_{g}^{\epsilon }$ values in bold underneath density plots are the calculated variances of these residuals. These gene families are sets of orthologs corresponding to the genes **a** *tatA*, **b** *devR*, **c** *waaW*, **d** *thrC*, **e** *gspA*, **f** *tssB*, **g** *dctS*, and **h** *ecnB*. Panels **a**,**b** show two invariable gene families with relatively high (**a**) and low (**b**) average abundance; similarly, panels **c**, **d** show two variable gene families with relatively low (**c**) and high (**d**) average abundances. Panels **e**, **f** show two gene families involved in secretion with similar abundances, but low (**e**) vs. high (**f**) variability. Finally, panels **g**, **h** show that both invariable (**g**) and variable (**h**) gene families can have substantial study-specific effects. (All gene families displayed were significantly (in)variable using CCoDA, FDR ≤5*%*)

This mean-variance relationship means that gene families encoding, for example, the bacterial ribosome, which are among the most abundant in these metagenomes, will therefore have the highest sample variance as well. Meanwhile, gene families with low average abundance, such as those involved in the disease signatures listed above, will appear to be invariable when in reality they are simply very infrequently observed. For example, three out of five of the invariable gene families annotated to pertussis only have one read each in a single sample, which constitutes extremely weak evidence for their presence in the metagenome, let alone invariability. This approach also leaves us unable to detect gene families that are variable but relatively abundant, as well as the opposite (Fig. 2 a–d).

Gene family abundances can also vary by study, because of both biological differences between populations and technical factors including library preparation, amplification protocol, and sequencing technology. However, gene families with large study effects may or not be variable within each study, and vice versa (see, e.g., Fig. 2 e–h). Our method should therefore also take this potential confounder into account.

Finally, to assess statistical significance, we need to assess the range of variances we would expect for a particular gene family given its mean abundance, requiring us to model the overall mean-variance relationship. Figure 1 shows that this mean-variance relationship cannot be adequately captured by a Poisson distribution (blue dashed line), in which the mean and variance are equal; however, a better fit can be obtained by using the negative binomial distribution (red dashed line), a count-based distribution that allows for overdispersion, i.e., variance that exceeds the mean. Indeed, simply based on this negative binomial best-fit, ribosomal proteins are likely less variable than expected since they fall well below the trend line in all three datasets (Fig. 1). The negative binomial is commonly used in other sequencing applications, such as RNAseq [21], which has similar overdispersion.

### A new test, CCoDA, captures the variability of microbial gene families

We present a model that enables gene family abundance to be quantitatively compared across metagenomes for thousands of microbial genes. To account for study effects, we fit a linear model of log abundance *D*_*g*,*s*_ for gene *g* in sample *s* as a function of the overall mean abundance *μ*_*g*_ and a term *β*_*g*,*y*_ that quantifies the offset for each study *y*:

1 $$ D_{g,s}=\mu_{g}+\sum_{y\in Y}I_{y,s}\beta_{g,y}+\epsilon_{g,s}  $$

where *I*_*y*,*s*_ is an indicator variable that is 1 if sample *s* belongs to study *y* and 0 otherwise. In this simple model, *β*_*g*,*y*_ is simply the mean of gene *g* in study *y* after subtracting the overall mean *μ*_*g*_, and *ε*_*g*,*s*_ are the residuals left after these study-specific means *β*_*g*,*y*_ are subtracted out.

The residual *ε*_*g*,*s*_ quantifies how much the abundance of gene *g* in sample *s* differs from the average abundance across samples in the same study as *s*. We denote the variance of the residuals across samples by $V_{g}^{\epsilon }$. When this statistic is small, the gene has similar abundance across samples after accounting for study effects. A large value of $V_{g}^{\epsilon }$ indicates that samples have very different abundances.

To assess the statistical significance of gene family variability, as suggested above, we compare the residual variance $V_{g}^{\epsilon }$ to a data-driven null distribution based on the negative binomial distribution (see the “Methods” section and Additional file 2: Figure S2). This approach is necessary because there is no straight-forward formula for the *p* value of $V_{g}^{\epsilon }$. Our method looks for deviations from the null hypothesis that gene families in the dataset have the same mean-variance relationship. This relationship is captured by the overdispersion parameters *k*_*y*_, such that the variance for a gene *g* in a study *y* is given by:

2 $$ \sigma^{2}_{g,y} = \beta_{g,y} + \frac{{\beta^{2}_{g,y}}}{k_{y}}  $$

where *β*_*g*,*y*_ are study-specific means for gene *g* as above.

Because this null distribution is generated stochastically per gene family from a count-based distribution matching the observed mean, i.e., by parametric bootstrapping, the null naturally accounts for the expected amount of noise based on the number of times a given gene family is observed. Gene families with low abundance or a high proportion of zeros are therefore more likely to be called non-significant than variable (Additional file 3: Figure S6 C–D).

We validated this approach further and assessed type I and type II error rates with simulated data (see the “Methods” section, Additional file 4: Figure S4), finding that CCoDA has high power and good control over the false positive rate when the overdispersion parameter *k* used in the null distribution is accurately estimated. To make the test more robust to factors affecting the estimation of *k* (Additional file 5: Figure S5), we also used simulation to control the false discovery rate empirically (Table 1).

**Table 1:** *q* value cutoffs to reach a given empirical FDR, estimated from simulation

Empirical FDR (%)	*q* value cutoff, variable	*q* value cutoff, invariable
5	0.0238	0.108
10	0.0669	0.180
25	0.181	0.294

CCoDA can be applied to shotgun metagenomes to sensitively and specifically identify variable genes in any environment without prior knowledge of factors that stratify relatively high versus low abundance samples.

### Thousands of variable gene families in the gut microbiome

Using CCoDA, we found 2357 gene families with more variability than expected and 5432 with less (leaving 9628 non-significant) at an empirical FDR of 5% (Additional file 3: Figure S6A). Restricting the analysis to gene families with at least one annotated representative from a bacterial or archaeal genome in KEGG, we obtained 1219 significantly variable and 3813 significantly invariable gene families (and 2194 non-significant). The differences in the residual variation of these gene families can be visualized using a heatmap of the residual *ε*_*g*,*s*_ values (Additional file 6: Figure S7 and Additional file 7: Figure S8). The large number of genes that were less variable than expected given their means supports the hypothesis of some functional redundancy in the gut microbiome, potentially due to selection for core functions that make microbes more successful in the gut environment. Notably, the HMP cohort tended to have overall lower variance in their metagenomes than the GC and T2D cohorts; this may be because the exclusion criteria for HMP, which explicitly studied only healthy individuals, were particularly stringent [41]. Nevertheless, our discovery of thousands of significantly variable genes across a range of abundance levels demonstrates that the gut microbiome is less invariable than prior work suggested.

This result highlights the importance of a quantitative, gene-level evaluation of functional stability. Importantly, the magnitude of the residual variance statistic $V_{g}^{\epsilon }$ is not the sole determinant of significance, as illustrated by the overlap in distributions of $V_{g}^{\epsilon }$ between the variable, invariable, and non-significant gene families. For example, both low-abundance gene families with many zero values and high-abundance but invariable gene families will tend to have low residual variance, but the evidence for invariability is much stronger for the second group. Our test accurately discriminates between these scenarios, tending to call the second group significantly invariable and not the first (Additional file 3: Figure S6A, inset), whereas an approach that simply thresholded $V_{g}^{\epsilon }$ would be unable to distinguish between them.

### Biological pathways contain both invariable and variable components

**Fig. 3:**
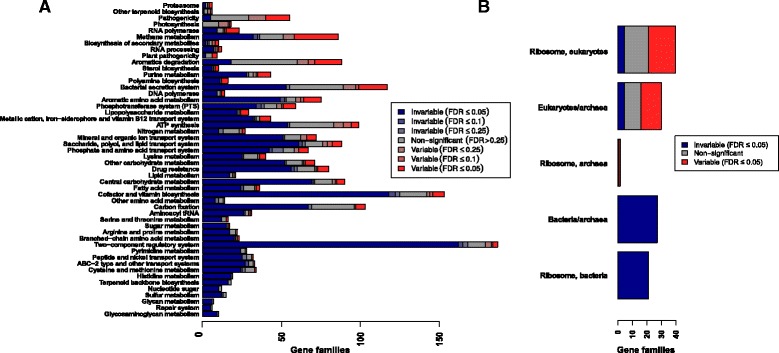
Most pathways include a mixture of both variable and invariable gene families. **a** Stacked bar plots show the fraction of invariable (*blue*), non-significant (*gray*), and variable (*red*) gene families annotated to KEGG Orthology pathway sets (*rows*), at different false discovery rate (FDR) cutoffs (color intensity). Only gene families with at least one annotated bacterial or archaeal homolog were counted. **b** Fraction of strongly invariable, non-significant, and strongly variable gene families within the ribosomes of different kingdoms. Row labels with only one kingdom indicate gene families unique to that kingdom, and rows with multiple kingdoms (e.g., “Eukaryotes/archaea”) indicate gene families shared between these two kingdoms. As expected, the bacterial ribosome was completely invariable

To test our hypothesis that the appearance of pathways and functional categories with similar abundance across samples can be explained by a subset of core components, we examined individual gene variability within KEGG modules. As expected, we observed an overall signal of stability at this broad level of gene groupings. Many of the pathways previously identified as invariable (e.g., aminoacyl-tRNA metabolism, central carbon metabolism) indeed have more invariable than variable genes. However, individual genes show a much more complex picture. Even the most invariable pathways also include significantly variable genes (Fig. 3). For example, the highly conserved KEGG module set “aminoacyl-tRNA biosynthesis, prokaryotes” included one variable gene at an empirical FDR of 5%, *sepRS*. *sepRS* is an O-phosphoseryl-tRNA synthetase, which is an alternative route to biosynthesis of cysteinyl-tRNA in methanogenic archaea [42]. Methanogen abundance has previously been noted to be variable between individual human guts: while DNA extraction for archaea may be less reliable than for bacteria, even optimized methods showed large standard deviations across individuals [43]. Another gene in this category was variable at a weaker level of significance (10% empirical FDR): *poxA*, a variant lysyl-tRNA synthetase. Recent experimental work has shown that this protein has a diverged, novel functionality, lysinylating the elongation factor EF-P [44, 45].

By comparison, 77% of the tested prokaryotic gene families in the KEGG module set “central carbohydrate metabolism” were significantly invariable, and 5.6% (five genes) were significantly variable (Additional file 8: Figure S9) at an empirical FDR of 5%. In this case, the variable gene families highlight the complexities of microbial carbon utilization (see Additional file 9 for details).

**Fig. 4:**
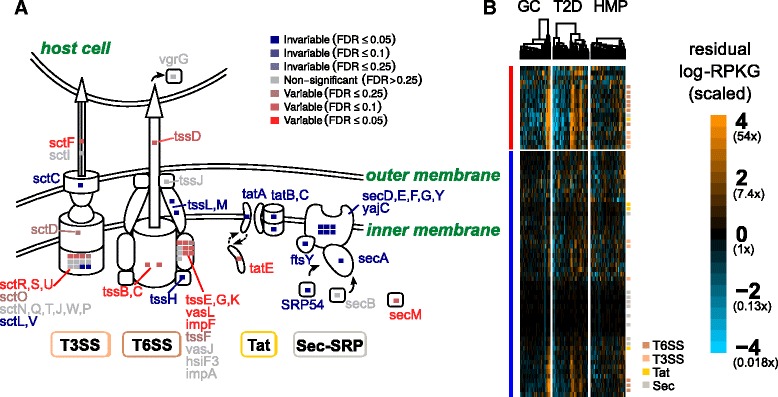
Variable and invariable gene families involved in bacterial secretion separate by gene function. **a** Schematic diagram showing the type III (T3SS), type VI (T6SS), Sec, and Tat secretion system gene families measured in this dataset. Gene families are color-coded by whether they were variable (*red*), invariable (*blue*), or neither (*gray*), with strength of color corresponding to the FDR cutoff (color intensity). *Insets* show a summary of how many gene families in KEGG modules corresponding to a particular secretion system were variable or invariable and at what level of significance. **b** Heatmaps showing scaled residual log-RPKG for gene families (*rows*) involved in bacterial secretion. Variable (*red*) and invariable (*blue*) gene families were clustered separately, as were samples within a particular study (*columns*). log-RPKG values were scaled by the expected variance from the negative binomial null distribution. Genes in specific secretion systems are annotated with *colored squares* (T6SS: *red-orange*; T3SS: *orange*; Tat: *yellow*; Sec: *grey*)

One of the more variable pathways was the “bacterial secretion system.” We found that the majority of significantly variable gene families annotated to this pathway (16 out of 18) were involved in specialized secretion systems, especially the type III and type VI systems (Fig. 4). These secretion systems are predominantly found in Gram-negative bacteria and are often involved in specialized cell-to-cell interactions, between microbes and between pathogens or symbionts and the host. They allow the injection of effector proteins, including virulence factors, directly into target cells [46, 47]. Type VI secretion systems are also determinants of antagonistic interactions between bacteria in the gut microbiome [48, 49].

In contrast, gene families in the Sec (general secretion) and Tat (twin-arginine translocation) pathways were nearly all significantly *invariable* at an empirical FDR of 5%, with only one gene in each being found to be significantly variable. This contradicts previous suggestions that the Sec and Tat pathways were some of the most variable in the human microbiome [13]. This discrepancy is probably due to our accounting for the mean-variance relationship in shotgun data. The Sec and Tat systems are abundant and phylogenetically diverse [50] and will therefore have greater variance than low-abundance genes just by chance. Our test adjusts for this feature of sequencing experiments and shows that these genes are in fact less variable than expected given their mean abundance.

Our results further demonstrate that analyzing functional variability at the level of pathways can obscure gene-family-resolution trends of potential biomedical importance. The variability of individual gene families involved in lipopolysaccharide (LPS) metabolism may exemplify such a case. LPS (also known as “endotoxin”) is a macromolecular component of the Gram-negative bacterial outer membrane, consisting of a lipid anchor called “lipid A,” a “core oligosaccharide” moiety, and a polysaccharide known as the “O-antigen” (which may be absent). Lipid A is sensed directly by the human innate immune system via the Toll-like receptor TLR4. Furthermore, lipid A variants with different covalent modifications (e.g., differentially acylated [51], phosphorylated [52], and palmitoylated [53] variants) have been shown to have different immunological properties (see Additional file 9: Supplementary information).

**Fig. 5:**
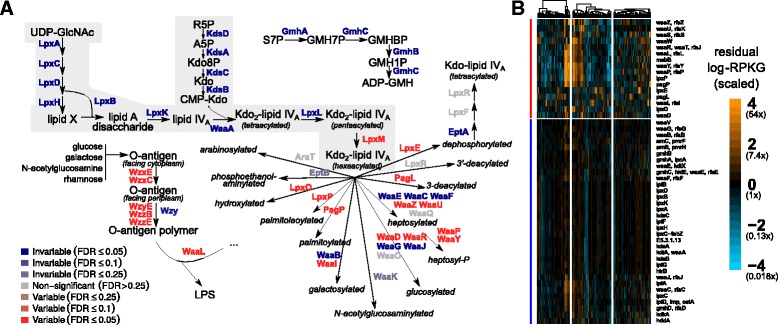
Central Kdo and lipid A biosynthesis is invariable, but many genes involved in covalent modifications to LPS are variable. **a** Pathway schematic showing a selection of measured gene families involved in lipopolysaccharide metabolism. Gene families are color-coded by whether they were variable (*red*) or invariable (*blue*), with strength of color corresponding to the FDR cutoff (color intensity). Central Kdo and lipid A metabolism is highlighted in *light gray*. Abbreviated metabolites are (*GlcNAc* N-acetylglucosamine), (*Kdo* ketodeoxyoctonate), (*R5P* ribose-5-phosphate), (*S7P* sedoheptulose-7-phosphate), (*GMH* glyceromannoheptose), (*aminoarabinose* 4-amino-4-deoxy-L-arabinose). **b** Heatmaps showing scaled residual log-RPKG for gene families (*rows*) involved in lipopolysaccharide metabolism, as in Fig. 4

We found that all but one gene family involved in the biosynthesis of lipid A, as well as all gene families involved in the biosynthesis of the core oligosaccharide components ketodeoxyoctonate (Kdo) and glyceromannoheptose (GMH), were significantly invariable (16 out of 17; Fig. 5). The lone exception catalyzes the the final lipid A acylation step, adding a sixth acyl chain; this gene family was significantly variable (FDR ≤ 5*%*). Furthermore, we observe several variable gene families annotated as performing covalent modifications of LPS, including hydroxyl- (*lpxO*), palmitoyl- (*pagP*), and palmitoleoylation (*lpxP*), as well as deacylation and dephosphorylation. These modifications can lead to differential TLR4 activation [53, 54]. We also observe that gene families involved in O-antigen synthesis and ligation to lipid A tended to be variable (5 out of 6). These results suggest that healthy individuals may differ in the amount of hexa- vs. pentaacylated LPS, and in the amounts of other LPS chemical modifications, and thus in their baseline level of TLR4-dependent inflammation. Importantly, since the majority of gene families annotated to LPS biosynthesis were invariable, this result would have been missed by considering the pathway as a unit.

### Many invariable gene families are deeply conserved

**Fig. 6:**
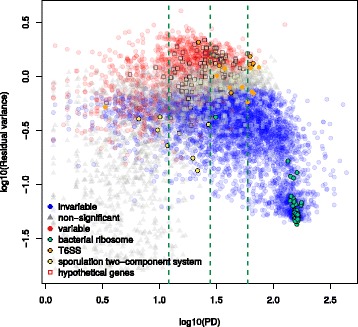
Phylogenetic distribution (PD) of gene families partially explains gene family variability. Scatter plot shows log10 PD (*x*-axis) vs. log10 residual variance statistic (*y*-axis). *Red points* were significantly variable and *blue points* were significantly invariable. Gene families in specific functional groups are also highlighted in different colors, specifically the bacterial ribosome (*green*), the type VI secretion system (or “T6SS”; *orange*), the KinABCDE-Spo0FA sporulation control two-component signaling system (*yellow*), and hypothetical genes (*tan squares*). Gene families that were significantly invariable (ribosome and sporulation control) or significantly variable (hypothetical genes and the T6SS) at an estimated 5% FDR are outlined in black. The bacterial ribosome, as expected, had very high PD and was strongly invariable. The type VI secretion system genes, in contrast, were conserved but variable, and some genes involved in the Kin-Spo sporulation control two-component signaling pathway had low PD but were invariable. Only gene families with at least one annotated bacterial or archaeal homolog are shown

Conservation of gene families across the tree of life is one factor we might expect to affect gene variability. For instance, ribosomal proteins should appear to be invariable merely because they are shared by all members of a given kingdom of life. To explore the relationship between gene family taxonomic distribution and variability in abundance across hosts, we constructed trees of the sequences in each KEGG family using ClustalOmega and FastTree. We then calculated phylogenetic distribution (PD), using tree density to correct for the overall rate of evolution (Dongying Wu, personal communication, 2015) (Fig. 6 a).

Overall, invariable gene families with below-median PD tended to be involved in carbohydrate metabolism and signaling. Specifically, these 2046 gene families were enriched for the pathways “two-component signaling” (*q*=1.5×10^−15^), “starch and sucrose metabolism” (*q*=1.8×10^−3^), “amino sugar and nucleotide sugar metabolism” (*q*=0.063), “ABC transporters” (*q*=2.4×10^−5^), and “glycosaminoglycan [GAG] degradation” (*q*=0.053), among others (Additional file 10). Enriched modules included a two-component system involved in sporulation control (*q*=0.018), as well as transporters for rhamnose (*q*=0.14), cellobiose (*q*=0.14), and *α*- and *β*-glucosides (*q*=0.14 and *q*=0.19, respectively). These results are consistent with the hypothesis that one function of the gut microbiome is to encode carbohydrate-utilization enzymes the host lacks [55]. Additionally, recent experiments showed that the major gut commensal *Bacteroides thetaiotaomicron*contains enzymes adapted to the degradation of sulfated glycans including GAGs [56, 57] and that many *Bacteroides* species can in fact use the GAG chondroitin sulfate as a sole carbon source [58].

Out of the 298 significantly variable gene families with the above median PD, we found no pathway enrichments but three module enrichments. These included the archaeal (*q*=1.5×10^−3^) and eukaryotic (*q*=8.7×10^−9^) ribosomes, which reflects differences in the relative abundance of microbes from these domains of life across hosts (Fig. 3 b). The third conserved but variable module was the type VI secretion system (*q*=0.039). Intriguingly, specialized secretion systems were also observed to vary within gut-microbiome-associated species in a strain-specific manner, using a wholly separate set of data [59]. Finally, gene families described as “hypothetical” were enriched in the high-PD but variable gene set (*p*=2.4×10^−8^, odds ratio = 2.2) and depleted in the low-PD but invariable set (*p*=5.4×10^−13^, odds ratio = 0.41).

Transporters show strain-specific variation in copy number across different human gut microbiomes [59], and analyses by Turnbaugh et al. identified membrane transporters as enriched in the “variable” set of functions in the microbiome [12]. However, we mainly found transporters enriched among gene families with similar abundance across hosts, despite being phylogenetically restricted (low-PD but invariable genes; Additional file 11). Part of this difference is likely due to our stratifying by phylogenetic distribution, a step previous studies did not perform.

### Proteobacteria are the major source of variable genes

**Fig. 7:**
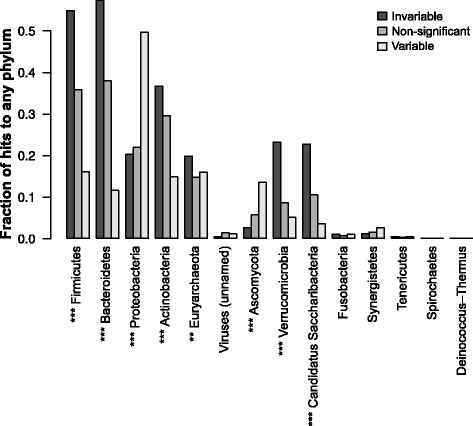
Variable gene families correlate with the predicted abundance of Proteobacteria. *Bar plots* give the fraction of gene families in each category (significantly invariable, non-significant, and significantly variable, 5% FDR) that were significantly correlated to predicted relative abundances of phyla, as assessed by MetaPhlAn2, using partial Kendall’s *τ* to account for study effects and a permutation test to assess significance. *Asterisks* give the level of significance by chi-squared test of non-random association between gene family category and the number of significant associations. (*** *p*≤10^−8^ by chi-squared test after Bonferroni correction; ** *p*≤10^−4^)

To assess which taxa contributed these variable and invariable genes, we first computed correlations between phylum relative abundances (predicted using MetaPhlAn2 [60]) and gene family abundances. Specifically, we used a permutation test based on partial Kendall’s *τ* correlation. This test is rank-based and thus distribution-agnostic, handles ties (unlike Spearman’s *ρ*), and accounts for study-to-study variation by using partial correlation (see the “Methods” section). The resulting *p* values were corrected for multiple testing using the *q* value method and thresholded at an FDR of *q*≤0.05. Based on these results, we then determined whether phyla were enriched for variable or invariable genes by Fisher’s exact test (Bonferroni-corrected *p*≤0.05). This analysis revealed that the predicted abundance of Proteobacteria was strongly enriched for correlations with variable gene families (Bonferroni-corrected *p*≤10^−8^): Fig. 7 b). The abundance of the archaeal phylum Euryarchaeota was also enriched for correlations with variable gene families, to a lesser extent (Bonferroni-corrected *p*≤10^−4^).

Proteobacteria were a comparatively minor component of these metagenomes (median = 1%), compared to Bacteroidetes (median=59*%*) and Firmicutes (median=33*%*: see Additional file 12: Figure S10), which were more associated with invariable genes (Bonferroni-corrected *p*≤10^−8^). Euryarchaeota comprised an even smaller fraction of the microbiome (median=0*%*) and was only detected in 33% of metagenomes (though this could potentially be explained by unreliable extraction efficiency for archaea, as mentioned above [43]). However, seven samples from the GC and T2D cohorts had ≥15% Proteobacteria, with four having ≥20% and one having 41%. Overgrowth of Proteobacteria has been associated with metabolic syndrome [32] and inflammatory bowel disease [61]. Also, Proteobacteria can be selected (over Bacteroidetes and Firmicutes) by intestinal inflammation as tested by TLR5-knockout mice [62], and some Proteobacteria can induce colitis in this background [63], potentially leading to a feedback loop. Thus, the variable gene families we discovered could be biomarkers for dysbiosis and inflammation in otherwise healthy hosts.

It has been proposed that a small number of “enterotypes” may exist in the human gut microbiome, each with distinct taxonomic composition [25, 26]. Most recently, abundances of the taxa *Ruminococcaceae*, *Bacteroides*, and *Prevotella* were found to explain the most taxonomic variation across individuals [28]. These enterotypes appeared to be linked to long-term diet, with *Prevotella* highest in individuals with the most carbohydrate intake and *Bacteroides* correlating with protein and animal fat. However, while these clades contributed most to taxonomic variation, in our study, all were actually *depleted* for associations with variable genes. In contrast, the Proteobacterial family *Enterobacteriaceae* (Additional file 13: Figure S12B), and to a lesser extent, Gammaproteobacteria in general (Additional file 13: Figure S12C) were much more likely to be associated with variable gene families. Similar results were also obtained using the centered log-ratio (clr) transform to correct potential compositionality artifacts (see Additional file 14: Figure S16). This suggests that compared to previously identified enterotype marker taxa, levels of Proteobacteria, and potentially Euryarchaeota, better explain person-to-person variation in gut microbial gene function. These less abundant phyla were missed in enterotype studies, likely because enterotypes were identified by methods that will tend to weight higher-abundance taxa more, and enterotypes were identified from taxonomic, not functional data.

Because Proteobacteria are a relatively well-annotated yet low-abundance phylum, we explored whether either of these characteristics explain their association with variable genes. Importantly, genes correlated with Actinobacteria did not tend to be variable, even though Proteobacteria and Actinobacteria had similar levels of abundance (Additional file 12: Figure S10). Also, while they were comparatively low abundance compared to Bacteroidetes or Firmicutes, Proteobacteria were also generally not close to the limit of detection when present: Proteobacterial relative abundance was more than 0.18 in 90% of samples, whereas MetaPhlAn2 was able to detect taxa with relative abundances of only 0.0004% in our data. Low abundance therefore does not appear to explain this association.

The number of phyla present in our data was not enough to determine whether there was any trend for low-prevalence or low-abundance taxa to be more correlated with variable genes. To answer this question, we conducted the same analysis with bacterial and archaeal taxa at the family level. However, when considering the 30 families with significant enrichments for (in)variable or non-significant gene families, there was no significant association between the degree of enrichment for variable genes and either prevalence (*r*=−0.07, *p*=0.72) or abundance (*r*=−0.1, *p*=0.58) (Additional file 13: Figure S12D-E). In fact, *Enterobacteriaceae*, a Proteobacterial family, was significantly enriched for correlations with variable genes despite a prevalence of 86%, in the top 25% of all families detected. Thus, prevalence and abundance do not explain the variability of Proteobacterial genes.

To investigate annotation bias, we first compared the numbers of genomes in KEGG for each phylum. There are 1111 Proteobacterial genomes compared to 575 for Firmicutes, 276 for Actinobacteria, 104 for Euryarchaeota, and only 97 for Bacteroidetes. Accordingly, Proteobacteria had the most gene families (1417) not annotated in any other phylum (“private” gene families), compared to 538 for Firmicutes, 342 for Euryarchaeota, 215 for Actinobacteria, and 21 for Bacteroidetes. Considering only these private gene families, Proteobacteria and Euryarchaeota were enriched for variable genes, as before, whereas variable genes were depleted in the other three phyla (Additional file 15: Figure S13A). This suggests that the level of annotation does not predict the amount of variable genes. In a further test, we repeated the entire statistical test on a subset of genes, sampling one part phylum-specific genes drawn equally from Proteobacteria, Actinobacteria, Firmicutes, and Euryarchaeota, and one part genes annotated to all four phyla (see the “Methods” section). Again, Proteobacteria- and Euryarchaeota-specific genes were significantly variable more often than those from either Actinobacteria or Firmicutes (Additional file 15: Figure S13B). We therefore concluded that phylum abundance and annotation bias do not explain the enrichment of variable genes in Proteobacteria.

Finally, variable genes also do not appear to be biomarkers for either taxonomic statistics or measured host characteristics. To explore this question, we used the same two-sided partial Kendall’s *τ* test as above. With regard to taxonomic statistics, we tested *α*-diversity (measured by Shannon entropy), the Bacteroidetes/Firmicutes ratio, and average genome size (AGS): however, all of these correlated more often with invariable gene families (see Additional file 9, Additional file 13: Figure S12A). For host characteristics, we selected body mass index, sex, and age, which were measured in all three studies we analyzed. None of these variables correlated significantly with *any* variable gene family abundances, even at a 25% false discovery rate.

One study (GC) measured blood levels of three inflammatory markers, TNF *α*, IL-1, and CD163, which did not correlate with Proteobacterial abundance in this study (Holm-corrected *p* value > 0.2, Kendall’s *τ*). However, other inflammatory markers directly linked to changes in Proteobacterial abundance (e.g., IgA, IL-10, and IL-17, reviewed in [32]) were not measured in this panel. These results suggest that major correlates of variation in microbiota gene levels, possibly including diet and specific inflammatory markers, remain to be measured.

### Bacterial phyla have unique sets of variable genes

**Fig. 8:**
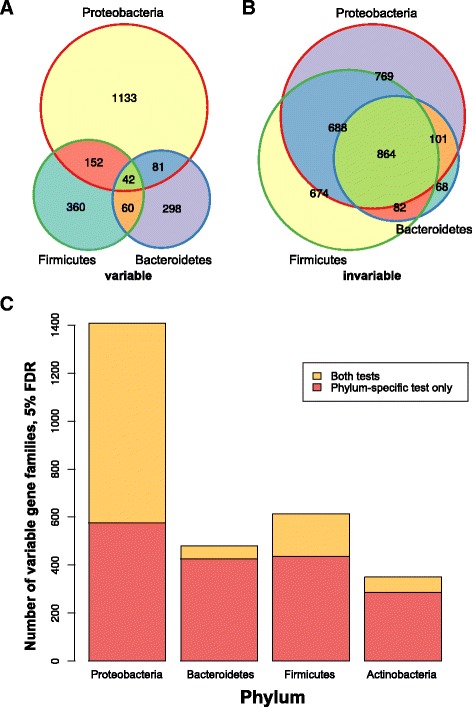
Phylum-specific tests reveal hidden variability in the most prevalent bacterial phyla. **a**, **b** Venn diagrams showing the number of significantly variable (**a**) and invariable (**b**) gene families across Proteobacteria, Bacteroidetes, and Firmicutes, FDR ≤5*%*. **c** *Bars* indicate the fraction of phylum-specific variable gene families that were also variable overall (*yellow*, “both tests”) or that were specific to a particular phylum (*red*, “phylum-specific test only”)

The variable gene families we identified seem to include both genes whose variance is explained by phylum-level variation (e.g., Proteobacteria) and genes that vary within fine-grained taxonomic classifications, such as strains within species. Also, some gene families may confer adaptive advantages in the gut only within certain taxa. To detect gene families that are variable or invariable within a phylum, we repeated the test, but using only reads whose best RAPSearch2 [64] alignments were to sequences from whole genomes of each of the four most abundant bacterial phyla (Bacteroidetes, Firmicutes, Actinobacteria, and Proteobacteria). Most (77%) gene families showed phylum-specific effects. Invariable gene families tended to agree, but the reverse was true for variable gene families: 19.4% of gene families that were invariable in one phylum were invariable in all, compared to just 0.34% (eight genes) in the variable set (Fig. 8 a, b). This trend was robust to the FDR cutoff (Additional file 16: Figure S14A–B). Gene families invariable in all four phyla were enriched for basal cellular machinery, as expected (Additional file 17: C–D).

The relationship between phylum-specific and overall gene family abundance variability differed by phylum. Proteobacteria-specific variable gene families tended to be variable overall (59%), whereas the proportions of gene families that were also variable overall were much lower for Bacteroidetes- (12%), Firmicutes- (29%), and Actinobacteria-specific (18%) gene families (Fig. 8 c). This supports the hypothesis that Proteobacterial abundance is a dominant factor influencing functional variability in the human gut microbiome. It further suggests that many overall-variable gene families are not only merely markers for the amount of Proteobacteria (or some other phylum) but are also variable at finer taxonomic levels, such as the species or even the strain level [59, 65].

Comparing the two dominant phyla in the gut, Bacteroidetes and Firmicutes, we further observe that the overall proportions of variable and invariable families were similar across pathways, with some interesting exceptions. For example, LPS biosynthesis had more invariable gene families in Bacteroidetes than in Firmicutes, which we expected given that LPS is primarily made by Gram-negative bacteria. Conversely, both two-component signaling and the PTS system had many more invariable gene families in Firmicutes than in Bacteroidetes (Additional file 16: Figure S14C). However, phylum-specific variable gene families tended not to overlap (median overlap, 0%, compared to 46% for invariable gene families). This was even true for pathways where the overall proportion of variable and invariable gene families was similar, such as cofactor and vitamin biosynthesis and central carbohydrate metabolism (Additional file 16: Figure S14D). Thus, unique genes within invariable pathways vary in their abundance across microbiome phyla.

Furthermore, the enriched biological functions of the phylum-specific variable gene families differed by phylum (Additional file 18). For instance, Proteobacterial-specific variable gene families were enriched (Fisher’s test enrichment *q*=0.13) for the biosynthesis of siderophore group nonribosomal peptides, which may reflect the importance of iron scavenging for the establishment of both pathogens (e.g., *Yersinia*) and commensals (e.g., *Escherichia coli*) [66]. Another phylum-specific variable function appeared to be the type IV secretion system (T4SS) within Firmicutes (*q*=0.021). Homologs of this specialized secretion system are involved in a wide array of biochemical interactions, including the conjugative transfer of plasmids (e.g., antibiotic-resistance cassettes) between bacteria [67]. We conclude that our approach enables the identification of substantial variation within all four major bacterial phyla in the gut, much of which is not apparent when data are analyzed at broader functional resolution or without stratifying by phylum.

## Discussion

This study presents a novel test for genes whose abundances are significantly more or less variable across individuals than expected. This test, which we call CCoDA, provides a finer resolution and more statistically grounded estimate of “functional redundancy” [68] than was previously possible in the human microbiome. CCoDA differs from earlier approaches to quantifying variability in microbiome function in several key ways. First, we focus explicitly on the variability of gene family abundance, not differences in mean abundance between predefined groups, as has been done to reveal pathways whose abundance differs between body sites [69] or disease states [6].

Second, by using a null distribution based on the negative binomial, our model accounts for stochastic variation in gene family abundance between individuals caused by sampling. This parametric bootstrap null is more computationally intensive than previous approaches. However, the use of such a null allows us much better control over the false discovery rate than previous approaches that dichotomized gene families based on binary presence/absence [12]. Dichotomizing in this way may be acceptable for small datasets. However, based on the data used here, dichotomizing would classify 12% of significantly invariable (FDR ≤0.05) gene families and, more problematically, 85% of non-significant gene families (q ≥0.25) as part of the “variable” metagenome. This problem is not easily avoided by picking a different presence/absence cutoff (see Additional file 19: Figure S15).

A third important aspect of our method is that the underlying model accounts for the mean-variance relationship in count data and corrects for systematic biases between studies. While estimating this mean-variance relationship accurately requires a significant sample size (the best results in simulations were obtained with *n*≥40 per study), CCoDA can identify individual gene families as well as pathways that break this overall trend. Because we account for the mean-variance relationship, we identify different variable pathways than the previous studies that relied on the sample variance only [13]. Additionally, our major findings are robust when we apply the centered log-ratio transform (see Additional file 14: Figure S16). Importantly, unlike previous work, CCoDA tends to call pathways that are well-conserved across prokaryotes invariable (for example, the Sec general secretory system; see Fig. 6). This suggests that this method better captures biological intuition about meaningful variation. Fourth, the null distribution is estimated from the shotgun data and does not require comparisons to sequenced genomes [12]. Finally, unlike previous approaches, CCoDA can be used for meta-analysis, integrating data from multiple different populations.

We found that basic microbial cellular machinery, such as the ribosome, tRNA-charging, and primary metabolism, were universal functional components of the microbiome, both in general and when each individual phylum was considered separately. This finding is consistent with the previous results [12] and indeed is not surprising given the broad conservation of these processes across the tree of life. In contrast, we identified invariable gene families that have narrower phylogenetic distributions. These included, for example, proteins involved in two-component signaling, starch metabolism (including glucosides), and glycosaminoglycan metabolism. Previous experimental work has underscored the importance of some of these pathways in gut symbionts: for instance, multiple gut-associated *Bacteroides* species use the glycosaminoglycan chondroitin sulfate as a sole carbon source [56], and the metabolism of resistant starch in general is thought to be a critical function of the omnivorous mammalian microbiome [55]. These results suggest that our method identifies protein-coding gene families that contribute to fitness of symbionts within the gut. Finally, we found a number of invariable gene families whose function is not yet annotated. These gene families may represent functions that are either essential or provide advantages for life in the gut and may therefore be particularly interesting targets for experimental follow-up (e.g., assessing whether strains in which these gene families have been knocked out in fact have slower growth rates, either in vitro or in the gut).

We also identified significantly variable gene families, including enzymes involved in carbon metabolism, specialized secretion systems such as the T6SS, and LPS biosynthetic genes. Proteobacteria, rather than Bacteroidetes or Firmicutes, emerge as a major source of variable genes, including some genes whose abundance also varied within the Proteobacteria (e.g., T6SS). Since Proteobacteria have been linked to inflammation and metabolic syndrome [32], we speculate that inflammation may be one variable influencing functions in the gut microbiome. Some variable genes, including many of unknown function, had surprisingly broad phylogenetic distributions.

Variable gene families have a variety of ecological interpretations, e.g., first-mover effects, drift, host demography, and selection within particular gut environments. Computationally distinguishing among these possibilities is likely to present challenges. For example, distinguishing selection from random drift will probably require longitudinal data and appropriate models. Separating effects of host geography, genetics, medical history, and lifestyle will be possible only when richer phenotypic data is available from a more diverse set of human populations. To control for study bias and batch effects, it will be important to include multiple sampling sites within each study.

While statistical tests focused on differences in variances are not yet common throughout genomics, there is recent precedent using this type of test to quantify the gene-level heterogeneity in single-cell RNA sequencing data [19, 20] and to identify variance effects in genetic association data [70]. Like Vallejos et al. [20], we model gene counts using the negative binomial distribution and identify both significantly variable and invariable genes. In contrast, we frame our method as a frequentist hypothesis test as opposed to a Bayesian hierarchical model. Our method also accounts for study-to-study variation. Also, unlike previous approaches in this domain, CCoDA does not require biological noise to be explicitly decomposed from technical noise. Thus, our method does not require the use of experimentally spiked-in controls, which are not present in most experiments involving sequencing of the gut microbiome. Instead, we detect differences from the average level of variability using a robust non-parametric estimator, which we show through simulation leads to correct inferences under reasonable assumptions.

Our null model does not explicitly account for zero-inflation, that is, the presence of more zeros than predicted by the negative binomial model; models incorporating zero-inflation have been proposed for taxonomic microbiome data [71–73]. However, only 1–2% of gene families showed significant zero-inflation, and our method tended to call these genes non-significant (Table 2). This suggests that zero-inflation may not be as severe a problem for measuring gene family abundance as it is for measuring microbial species. However, if applied to a dataset where measurements were expected to be more sparse, the method could be modified to generate the null from a zero-inflated negative binomial distribution.

**Table 2:** Number of genes (with at least one bacterial/archaeal representative) with significant zero-inflation in each dataset, *q*≤0.05

		Glucose control	Type II diabetes	Human microbiome project
Invariable (5% FDR)	Inflated	16	42	34
	Total	3768	3768	3768
Variable (5% FDR)	Inflated	6	11	21
	Total	1218	1218	1215
Non-significant	Inflated	55	67	72
	Total	2161	2151	2117

A statistical method for detecting significant (in)variability similar to the one we present here could also be applied to other biomolecules measured in counts, such as metabolites, proteins, or transcripts. Performing such analyses on human microbiota would reveal patterns in the variability in the usage of particular genes, reactions, and pathways, which would expand on our investigation of potential usage based on presence in the DNA of organisms in host stool. Integrating the results of these analyses could also further help to validate or interpret the functional variability we observe in this dataset. For example, mass spectrometry methods that can resolve differently modified LPS molecules could reveal whether the variation we observe at the metagenomic level is also seen across LPS molecules with different immunogenic properties. Of course, we would also expect that key functions provided by the microbiome would be highly regulated at the level of transcript or protein abundance. Integrating transcript and/or protein variability with DNA variability would allow us to come up with more precise hypotheses about which functions are effectively constitutive and which are more strongly modulated by the gut environment.

Another important extension will be to generalize our method for comparing hosts from different predefined groups (e.g., disease states, countries, diets) to identify gene families that are invariable in one group (e.g., healthy controls) but variable in another (e.g., patients), analogously to recent methods for the analysis of single-cell RNAseq [21] and GWAS [70] data. In particular, gene families whose variance differs between case and control populations could point to heterogeneity within complex diseases, interactions between the microbiome and latent variables (e.g., environmental or genetic), and/or differences in selective pressure between healthy and diseased guts. Investigating group differences in functional variability could thereby allow the detection of different trends from the more common comparison of means.

## Conclusions

We present a statistical test for variability called CCoDA that can integrate data from multiple studies to identify individual variable and invariable gene families. Simulations reveal CCoDA has high accuracy and power across a range of realistic scenarios. Applying this test to shotgun metagenomes from healthy human gut microbiota, we uncovered thousands of variable gene families whose abundances were more variable than expected. In general, more conserved genes tended to be less variable, but significantly variable genes also included some with relatively broad phylogenetic distributions. Finally, while the phyla Bacteroidetes and Firmicutes varied substantially between healthy individuals, consistent with previous studies of the human gut microbiome, we found that these phyla were actually depleted for associations with variable genes. The same was true for genera and families used to define “enterotypes.” Instead, a less abundant phylum, Proteobacteria, contributed most to functional variation in this population. These results argue that gene function in the healthy human gut microbiome may be more variable than previously assumed and that the major axes of taxonomic variation in microbiota do not necessarily capture the most variation in function.

## Methods

### Overview

CCoDA takes as input reads that have been mapped to a reference library of gene families, yielding counts of gene families in each sample (see “Data collection and processing” in the “Methods” section). The following general process is then applied (see also Additional file 2: Figure S2 for a graphical depiction):

- Counts are normalized for genome size and gene length, yielding reads per kilobase of genome equivalent (RPKG) (the “Data normalization” section)
- Confounding factors, like study-to-study variation, are regressed out using a linear model (the “Model fitting to correct for covariates” section)
- The variance of the resulting residuals is calculated per gene (the “Model fitting to correct for covariates” section);
- A null distribution is generated (the “Modeling residual variances under the null distribution” section):
  - An overdispersion parameter *k*_*y*_ giving the mean-variance relationship is fit (per study *y*)
  - This parameter, along with the estimated means of each gene, is used to generate null count data via parametric bootstrap
  - The first four steps are repeated on the null count data to obtain null residual variances for each gene
  - Repeat until the desired number of bootstrap samples is reached
- Based on the resulting null distribution, *p*-values are calculated and corrected for multiple testing.

### Data collection and processing

Stool metagenomes from healthy human guts were obtained from three sources:

1. Two American cohorts from the Human Microbiome Project [13], *n*=42 samples selected,
2. A Chinese cohort from a case-control study of type II diabetes (T2D) [33], *n*=44 samples from controls with neither type II diabetes nor impaired glucose tolerance, and
3. A European cohort from a case-control study of glucose control [34], *n*=37 samples from controls with normal glucose tolerance.

These studies were chosen because they contained large cohorts of healthy individuals and were publicly available at the time at which we began this study. Samples (see Additional file 20 for SRA IDs) were chosen to have at least 1.5×10^7^ reads and mode average quality scores ≥20 (estimated via FastQC [74]). For consistency, each sample was rarefied to a depth of 1.5×10^7^ reads, and as reads from HMP were particularly variable in length, they were trimmed to a uniform length of 90 bp.

After downloading these samples from NCBI’s Sequence Read Archive (SRA), the FASTA-formatted files were mapped to KEGG Orthology (KO) [75] protein families with ShotMAP [35], an algorithm based on the aligner RAPSearch2 [64]. Bit-score cutoffs for matching a particular protein family were selected based on the average read length of each sample as described [35]. The KEGG Orthology database was chosen because it annotates a large number of bacteria and archaea, including many species observed in the human gut, and covers a wide range of gene families, including metabolic enzymes, signaling proteins, and virulence factors.

### Data normalization

The gene family counts were normalized for two confounders:

1. Average family length (AFL) or the average length of the matched genes within a gene family
2. Average genome size (AGS) or the estimated average genome length based on single-copy universal marker genes (estimated using MicrobeCensus: [36] http://github.com/snayfach/MicrobeCensus).

Normalization for these two factors yielded abundance values in units of RPKG or reads per kilobase of genome equivalents [36].

These RPKG abundance values were strictly positive with a long right tail and highly correlated with the variances (Spearman’s *r*=0.99). This strong mean-variance relationship is likely simply because these abundances are derived from counts that are either Poisson or negative binomially distributed. We therefore took the natural log of the RPKG values as a variance-stabilizing transformation. Because log0 is infinite, we added a pseudocount before normalizing the counts and taking the log transform. Since there is no AFL when there are no reads for a given gene family in a given sample, we imputed it in those cases using the average AFL across samples.

### Model fitting to correct for covariates

We fit a linear model to the data matrix of log-RPKG *D* of log-RPKG described above, with *n* gene families by *m* samples. The purpose of this linear model is to regress out variation caused by factors we were not interested in (here, study-to-study variation and per-gene-family mean values):

3 $$ D_{g,s}=\mu_{g}+\sum_{y\in Y}I_{y,s}\beta_{g,y}+\epsilon_{g,s}  $$

where *g*∈ [ 1,*n*] is a particular gene family, *s*∈ [ 1,*m*] is a particular sample, *μ*_*g*_ is estimated by the grand (i.e., overall) mean of log-RPKG $\frac {\sum _{s}D_{g,s}}{m}$ for a given gene family *g*, *Y* is the set of studies, *I*_*y*,*s*_ is an indicator variable valued 1 if sample *s* is in study *y* and 0 otherwise, *β*_*g*,*y*_ is a mean offset for gene family *g* in study *y*, and the residual for a given gene family and sample are given by *ε*_*g*,*s*_. For each gene family, the variance across samples of these *ε*_*g*,*s*_, which we term the “residual variance” or $V_{g}^{\epsilon }$, was our statistic of interest. In this case, residuals can be obtained simply by subtracting the per-dataset means from each gene family.

Overall trends in these data are explained well by this model, with an *R* ^2^=0.20. The residuals, which are approximately symmetrically distributed around 0, represent variation in gene abundance not due to study effects.

### Modeling residual variances under the null distribution

Having calculated this statistic $V_{g}^{\epsilon }$ for each gene family *g*, we then needed to compare this statistic to its distribution under a null hypothesis *H*_0_. This required us to model what the data would look like if in fact there were no surprisingly variable or invariable gene families. To do this, we used the negative binomial distribution to model the original count data (before adding pseudocounts and normalization to obtain RPKG).

The negative binomial distribution is commonly used to model count data from high-throughput sequencing. It can be thought of as a mixture of Poisson distributions with different means (themselves following a Gamma distribution). Like the Poisson distribution, the negative binomial distribution has an intrinsic mean-variance relationship. However, instead of a single parameter controlling both mean and variance as in the Poisson, the negative binomial has two, a mean parameter *μ* and a “size” or “overdispersion” parameter *k*. *k* is defined by $k=\frac {\mu ^{2}}{\sigma ^{2}-\mu }$. (If the sample mean is plugged into *μ* and the sample variance into *σ* ^2^, this equation also gives a method-of-moments estimator for *k*.) *k* ranges from (0,*∞*), with smaller values corresponding to more overdispersion (i.e., higher variance given the mean) and larger values approaching, in the limit, the Poisson distribution.

To model the case where no gene family has unusual variance given its mean value (i.e., our null hypothesis), we assumed that the data were negative binomially distributed with the observed means *μ*_*g*,*y*_ for each gene *g* and study *y*, but where the amount of overdispersion was modeled with a single size parameter *k*_*y*_ for each study *y*. This has similarities to previous approaches to model RNAseq distributions [22, 39, 76] and to identify (in)variable genes from single-cell RNAseq data [20] (see also the “Discussion” section).

$$\begin{array}{cc} H_{0}: & V_{g}^{\epsilon}=V_{g}^{\epsilon}\vert D_{g,s}\sim NB\left(\mu_{g,y},\:k_{y}\right)\\ H_{\text{alt}}: & V_{g}^{\epsilon}\neq V_{g}^{\epsilon}\vert D_{g,s}\sim NB\left(\mu_{g,y},\:k_{y}\right) \end{array} $$

To estimate this $\widehat {k_{y}}$, the overall size parameter for a given study *y*, we first calculated a $\widehat {k}$ value for every gene in that study with the method-of-moments estimator from above, then estimated the mode of these individual $\widehat {k_{g,y}}$ values. We estimated the mode by fitting a Gaussian kernel density estimate to the log-transformed $\widehat {k_{g,y}}$ values, and then finding the $\widehat {k_y}$ value that gave the highest density. (From simulations, we found that the mode method-of-moments was more robust than the median or harmonic mean; see Additional file 21: Figure S3. We use the harmonic mean here because the arithmetic mean of $\widehat {k_{g,y}}$ is highly unstable, probably because the distribution of $\widehat {k}$ has a long right-hand tail [77]).

Having estimated $\widehat {k_y}$ and the per-gene means $\widehat {\mu _g}$, we can now easily generate count data under this null distribution, yielding a parametric bootstrap null. These null count data are then treated identically to the real data: we add a pseudocount and normalize by AFL and AGS, fit the above linear model, and obtain null residual variances $V_{g}^{\epsilon _{0}}$ exactly as before.

Once the null is generated, statistical significance was obtained by a two-tailed test:

$$p_{g}=\frac{\#\left(\left(\frac{V_{g}^{\epsilon_{0}}-\overline{V_{g}^{\epsilon_{0}}}}{\overline{V_{g}^{\epsilon_{0}}}}\right) ^{2}\ge\left(\left(\frac{V_{g}^{\epsilon}-\overline{V_{g}^{\epsilon_{0}}}}{\overline{V_{g}^{\epsilon_{0}}}}\right)^{2}\right)\right)+1}{B+1} $$

Here, *B* refers to the number of null test statistics $V_{g}^{\epsilon _{0}}$ (in this case, *B*=750), and the overlined test statistics refer to their mean across the null distribution.

The resulting *p* values were then corrected for multiple testing by converting to FDR *q*-values using the procedure of Storey et al. [78] as implemented in the qvalue package in R [79].

An alternative approach to determining significance is based on the bootstrap. While using a parametric null distribution allows us to explicitly model the null hypothesis, it also breaks the structure of covariance between gene families, which may be substantial because genes are organized into operons and individual genomes within a metagenome. This structure can, optionally, be restored using a strategy outlined by Pollard and van der Laan [80]. Instead of using the test statistics $V_{g}^{\epsilon _{0}}$ obtained under the parametric null as is, we can use these test statistics to center and scale non-parametric bootstrap test statistics $V_{g}^{\epsilon \prime }$, which we derive from applying a cluster bootstrap with replacement from the real data and then fitting the above linear model (3) to the resampled data to obtain bootstrap residual variances:

$$V_{g}^{\epsilon_{0}\prime}=\left(\left(\frac{V_{g}^{\epsilon\prime}-\overline{V_{g}^{\epsilon\prime}}} {sd\left(V_{g}^{\epsilon\prime}\right)}\right)\times sd\left(V_{g}^{\epsilon_{0}}\right)\right)+\overline{V_{g}^{\epsilon_{0}}} $$

A similar non-parametric bootstrap approach has previously been successfully applied to testing for differences in gene expression [80].

### Visualization

As expected, when the residuals are plotted in a heatmap as in Additional file 6: Figure S7, variable gene families were generally brighter (i.e., more deviation from the mean) than invariable gene families, though not exclusively: this is because our null distribution, unlike the visualization, models the expected mean-variance relationship. We visualized this information by scaling each gene family by its expected standard deviation under the negative binomial null (i.e., by the mean root variance $\sum _{b\in [1,B]}\sqrt {V_{g_{b}}^{\epsilon _{0}}}/B$) (Additional file 7: Figure S8).

In Fig. 4, for comparability with existing literature, gene families in the T6SS were named by mapping to the COG IDs used in Coulthurst [47], except when multiple KOs mapped to the same COG ID; in these cases, the original KO gene names were kept. Schematics of the T3SS, T6SS, Tat, and Sec pathways were modeled on previous reviews [47, 81, 82] and on the KEGG database [75]. The pathway diagram in Fig. 5 is based on representations in the KEGG database [75], MetaCyc [83], and reviews by Wang and Quinn [84] and Whitfield and Trent [85]. These reviews were also used to identify KEGG Orthology gene families that were involved in lipopolysaccharide metabolism but not yet annotated under that term.

### Power analysis

Statistical tests should have reasonable power (also called “recall”) and control *α*, the false positive or type I error rate, at the desired level (e.g., 5% for a *p* value cutoff of 0.05). Our test controls *α* as expected if the correct size parameter *k* is estimated from the data (Additional file 21: Figure S3a-b). Estimating this parameter accurately is difficult, however, particularly for highly over-dispersed data [77], and in this case, we must also estimate this parameter from a mixture of true positives and nulls. We found that the mode of per-gene-family method-of-moments estimates was more robust to differences in the ratio of variable to invariable true positives (Additional file 21: Figure S3e–g) than the median or harmonic mean (the harmonic mean mirrors the approach in Yu et al. [76]).

Power analysis was performed on simulated datasets comprising three simulated studies. For each study, 1000 gene families were simulated over *n*∈{60, 120, 480, 960} samples. Null data were drawn from a negative binomial distribution with a randomly selected size parameter *k* common to all gene families, which was drawn from a log-normal distribution (log-mean =−0.65, sd =0.57). Gene family means were also drawn from a log-normal (log mean =2.94, sd =2.23). True positives were drawn from a similar negative binomial distribution, but where the size parameter was multiplied by an effect size *z* (for variable gene families) or its reciprocal 1/*z* (for invariable gene families). The above test was then applied to the simulated data, and the percents of type I and II errors (i.e., false positive and false negatives) were calculated by comparing to the known gene family labels from the simulation. Using similar parameters to those estimated from our real data, we saw that *α* decreased and power approached 1 with increasing sample size (see Additional file 4: Figure S4) and that *n*=120 appeared to be sufficient to achieve control over *α*.

### Calculation of an empirical FDR

At *n*=120, we also noted that *α* appeared to be greater for variable vs. invariable gene families (Additional file 5: Figure S5). This could be because accurately detecting additional overdispersion in already over-dispersed data may be intrinsically difficult. Instead of using a single *q* value cutoff for both variable and invariable genes, we performed additional simulations to determine what *q* value cutoff corresponded to an empirical FDR of 5%. We calculated appropriate cutoffs based on datasets with 43% true positives and a variable to invariable gene family ratio ranging from 0.1 to 10, taking the median cutoff value across these ratios (Additional file 10). Using these cutoffs, the overall dataset had 45% true positives and a variable to invariable gene family ratio of 0.43, indicating that these simulations were realistic.

### Estimating the phylogenetic distribution of gene families

To obtain estimates of the PD of KO gene families, we first obtained sequences of each full-length protein annotated to a particular KO. These sequences were then aligned using ClustalOmega [86]. The resulting multiple alignments were then used to generate trees via FastTree [87]. For both the alignment and tree building, we used default parameters for homologous proteins.

For all gene families represented in at least five different archaea and/or bacteria (6703 families total), we then computed tree densities, or the sum of edge lengths divided by the mean tip height. Using tree density instead of tree height as a measure of PD corrects for the rate of evolution, which can otherwise cause very highly conserved but slow-evolving families like the ribosome to appear to have a low PD (Dongying Wu, personal communication, 2015). Empirically, this measure is very similar to the number of protein sequences (Additional file 22: Figure S11) but is not as sensitive to high or variable rates of within-species duplication: for example, families such as transposons, which exhibit high rates of duplication as well as copy number variation between species, have a larger number of sequences than even very well-conserved proteins such as RNA polymerase, but have similar or even lower tree densities, indicating that they are not truly more broadly conserved.

Many protein families (8931 families) did not have enough observations to reliably calculate tree density, with almost all of these being annotated in only a single bacterial/archaeal genome. For these, we predicted their PD by extrapolation. To predict PD, we used a linear model that predicted tree density based on the total number of annotations (including annotations in eukaryotes). In fivefold cross-validation, this model actually had a relatively small mean absolute percentage error (MAPE) of 13.1%. We also considered a model that took into account the taxonomic level (e.g., phylum) of the last common ancestor of all organisms in which a given protein family was annotated, but this model performed essentially identically (MAPE of 13.0%). Predicted tree densities are given in Additional file 23. The PD of gene families varied from 1.2 (an iron-chelate-transporting ATPase only annotated in *Helicobacter pylori*) to 434.9 (the *rpoE* family of RNA polymerase sigma factors).

### Gene family enrichment

We were interested in whether particular pathways were enriched in several of the gene family sets identified in this work. For subsets of genes (such as those with specifically low PD), a two-tailed Fisher’s exact test (i.e., hypergeometric test) was used instead to look for cases in which the overlap between a given gene set and a KEGG module or pathway was significantly larger or smaller than expected. The background set was taken to be the intersection of the set of gene families observed in the data with the set of gene families that had pathway- or module-level annotations. *p* values were converted to *q* values as above. Finally, enrichments were enumerated by selecting all modules or pathways below *q*≤0.25 that had positive odds ratios (i.e., enriched instead of depleted).

### Associations with clinical and taxonomic variables

We used a general, non-parametric approach to detect association of residual RPKG with clinical and taxonomic variables (e.g., the inferred abundance of a particular phylum or other clade via MetaPhlAn2). To take into account potential study effects in clinical and taxonomic variables without using a parametric modeling framework, we used partial Kendall’s *τ* correlation as implemented in the ppcor package for R [88], coding the study effects as binary nuisance variables.

Kendall’s *τ* was used instead of Spearman’s *ρ* because while both are correlations based on ranks, Kendall’s *τ* performs better when many observations have the same rank. This is a particular problem with taxonomic data because many taxa have zero abundance in some samples, making their ranks equal.

The null distribution was obtained by permuting the clinical/taxonomic variables within each study 250 times and then re-assessing the partial *τ*. Finally, *p* values were calculated by taking the fraction of null partial correlations equally or more extreme (i.e., distant from zero) than the real partial correlations.

Taxonomic relative abundances were predicted from the shotgun data by MetaPhlAn2 with the very sensitive flag [60].

Two approaches were used to test for annotation bias. First (Additional file 15: Figure S13A), gene families private to a phylum (i.e., those annotated in only a single bacterial/archaeal phylum) were identified from the KEGG database. We then tested whether these private gene families were enriched or depleted for significantly variable gene families (5% FDR) using Fisher’s exact test. Second (Additional file 15: Figure S13B), we performed a test in which we sampled 215 private gene families from each of Proteobacteria, Firmicutes, Actinobacteria, and Euryarchaeota, totaling 860, plus 860 gene families annotated in all four phyla. (Since Bacteroidetes only had 21 private genes, that phylum was dropped from this analysis.) Enrichment/depletion for variable gene families within each phylum was performed as above.

### Phylum-specific tests

We created taxonomically restricted datasets in which the abundance of each gene family was computed using only metagenomic reads aligning best to sequences from each of the four most abundant bacterial phyla (Bacteroidetes, Firmicutes, Actinobacteria, and Proteobacteria). Phylum-specific data were obtained from the overall data as follows. First, the NCBI taxonomy was parsed to obtain species annotated below each of the four major bacterial phyla (Bacteroidetes, Firmicutes, Actinobacteria, and Proteobacteria); these species were then matched with KEGG species identifiers. Next, the original RAPSearch2 [64] results were filtered, so that the only reads remaining were those for which their “best hit” in the KEGG database originally came from the genome of a species belonging to the specific phylum in question (e.g., *E. coli* for Proteobacteria).

Since estimates of average genome size made from the entire metagenome might differ from estimates made on specific clades only, when performing the test, we normalized for AGS by dividing gene family counts by the median abundance of a set of 29 bacterial single-copy marker gene families [37]. These gene families were filtered in the same phylum-specific way as all other gene families. This approach is similar to the MUSiCC method for average genome size correction [89] and also controls for overall changes in phylum abundance. We also corrected for AFL as above.

Finally, we estimated the average level of overdispersion $\widehat {k_{y}}$ for individual studies based on the full dataset (not phylum-restricted). We took this approach because the expectation that <50% of gene families were differentially variable might not hold within each individual phylum. This could happen if, for example, different phyla had larger or smaller “core” genomes or were more or less prone to taking up DNA from the environment. We used the same *q* value cutoffs as in the overall test to set an estimated empirical FDR (Table 1). Otherwise, tests were performed as above.

### Zero inflation

Zero inflation was assessed separately for each gene in each dataset by fitting the observed counts to a zero-inflated model (using the zeroinfl function in the R package pcsl [90, 91]) and testing significance of the zero-inflation term. If the observed counts did not contain any zeros, the *p* value was assumed to be 1. *p* values were converted to *q* values as above to correct for multiple testing.

### Figures

Source data used to create main-text figures is provided in Additional file 24.

## Additional files

Additional file 1 **Figure S1.** The mean-variance relationship does not depend on the total number of samples. The glucose control (GC) study (*n*=37) was subsampled to various numbers of samples (9, 12, 18, 28), and the means, variances, and best-fits were computed as in Fig. 1, showing that this relationship is highly robust to sample size. (PDF 4298 kb)

Additional file 2 **Figure S2.** Schematic shows overview of data processing and method. (A) Data were collected from multiple datasets, mapped using Shotmap [35] and normalized for average genome size [36] and average gene family length. (B) The test integrates multiple studies using a linear model, then uses a parametric bootstrap to generate the null distribution for this linear model’s residual variance. See Additional file 9 for a full description. (PDF 57 kb)

Additional file 3 **Figure S6.** We identified significantly variable and invariable gene families, which are not explained by means near the limit of detection or by large numbers of zeros. (A) Density plots of distributions of residual variance (*V*_*G*_) statistics for significantly invariable (blue dashed line), non-significant (black solid line), and significantly variable (red dashed line) gene families. The distributions had the expected trend (e.g., significantly variable gene families tended to have higher residual variance) but also overlapped, indicating the importance of the calculated null distribution. The inset shows the proportion of zero values for the non-significant (black) and significantly invariable (blue) gene families with *V*_*G*_ falling in the lowest range (vertical dashed lines), indicating that the test differentiates between gene families that only appear invariable because they have few observations and gene families that are consistently abundant yet invariable. (B-C) Density plots of distributions of log10 mean counts (B) and fraction of zeros (C) across all three datasets for significantly invariable (blue dashed line), non-significant (black solid line), and significantly variable (red dashed line) gene families. Invariable gene families are not shown on the right because they overwhelmingly have small numbers of zeros. Gene families with very low mean abundances or large numbers of zeros tend to be called non-significant, not variable, indicating that the test correctly accounts for stochastic noise from low numbers of observations in determining statistical significance. (PDF 186 kb)

Additional file 4
**Figure S4.** Size parameter estimation affects power and *α*, with the mode method-of-moments giving the best control. *α* (A) was minimized and power (B) was maximized when the mode method-of-moments estimator was used to get estimates of the study-specific dispersion parameters $\widehat {k_{y}}$. Bars are from four simulations. The proportion of variable/invariable gene families was 0.4, and 43% of genes were true positives. (PDF 44 kb)

Additional file 5 **Figure S5.** The mode estimator is robust to changes in the proportion of true positives and the ratio of variable to invariable gene families. *α* (A-C) and power (D-F) as a function of the proportion of true positives (*x*-axis) and the ratio of variable to invariable true positives (*y*-axis) for *n*=120. *α*=0.05 and power =1 are shown in color-bars to the left of each heatmap for reference. *α* and power were calculated overall (left), for variable gene families (center), and for invariable gene families (right). In general, *α* was better controlled for the invariable gene families than for the variable gene families; we therefore used different empirical cutoffs for each set of genes. (PDF 131 kb)

Additional file 6 **Figure S7.** Heatmap showing significantly variable and invariable gene families (unscaled). Heatmap showing residual log-RPKG abundances (i.e., after normalizing for between-study effects and gene-specific abundances) of significantly invariable (blue) and significantly variable (red) gene families. Variable and invariable gene families were clustered separately, while samples were clustered within each dataset. (PDF 158 kb)

Additional file 7 **Figure S8.** Heatmap showing significantly variable and invariable gene families (scaled). As with Additional file 6: Figure S7, but residual log-RPKG abundances were scaled by their expected variance under the negative binomial null model (see the “Methods” section). (PDF 161 kb)

Additional file 8 **Figure S9.** Carbon metabolism contains variable and invariable gene families. (A) Pathway schematic showing a selection of measured gene families involved in central carbohydrate metabolism. Gene families are color-coded by whether they were variable (red) or invariable (blue), with strength of color corresponding to the FDR cutoff (color intensity). Genes involved in the Entner-Doudoroff pathway (*edd*), pentose metabolism (*fae-hps*), hexose metabolism (K01622, K16306), and tricarboxylic acid cycle intermediate metabolism (*frdCD*) were variable across healthy hosts. Abbreviated metabolites are glucose-6-phosphate (G6P), fructose-6-phosphate (F6P), fructose-1,6-bisphosphate (FBP), glyceraldehyde-3-phosphate (GAP), dihydroxyacetone phosphate (DHAP), 6-phosphogluconolactone (6PGL), 6-phosphogluconate (6PG), 2-keto-3-deoxy-phosphonogluconate (KDPG), ribulose-5-phosphate (R5P), ribose-5-phosphate (R5P), pyruvate (pyr), hexulose-6-phosphate (Hu6P), formaldehyde (HCHO), 2-amino-3,7-dideoxy-D-threo-hept-6-ulosonate (ADTH), and tetrahydromethanopterin (H_4_MPT). B) Heatmaps showing scaled residual log-RPKG for gene families (rows) involved in central carbohydrate metabolism. Variable (red) and invariable (blue) gene families were clustered separately, as were samples within a particular study (columns). log-RPKG values were scaled by the expected variance from the negative-binomial null distribution. (PDF 248 kb)

Additional file 9 Supplementary information. (PDF 98 kb)

Additional file 10 Module and pathway enrichments for variable and invariable gene sets (Fisher’s exact test q ≤ 0.25). (CSV 7 kb)

Additional file 11 Module and pathway enrichments for variable/high-PD and invariable/low-PD gene sets (Fisher’s exact test *q*≤0.25). (CSV 2 kb)

Additional file 12
**Figure S10.** Violin plots showing distributions of abundant phyla. (A) Abundance and (B) logit-transformed abundance ($\log {(\frac{a}{1-a}+10^{-6})}$, where 10^−6^ was added to prevent taking the log of zero) distributions were plotted for the six most abundant phyla. (PDF 155 kb)

Additional file 13 **Figure S12.** Variable gene families are less-often correlated to measured host characteristics or enterotype-associated taxa and are more often correlated to Proteobacterial clades. (A-C) Bar plots give the fraction of gene families with at least one bacterial or archaeal representative in each category (significantly invariable, non-significant, and significantly variable) that were significantly correlated to various sample characteristics or taxonomic abundances, using partial Kendall’s *τ* to account for study effects and a permutation test to assess significance. (A) Fraction correlating (*q*≤0.05) to average genome size (AGS), the ratio of Bacteroidetes to Firmicutes (B/F ratio), and a measure of *α*-diversity (Shannon index). (B) Fraction correlating (*q*≤0.05) to the predicted abundance of specific bacterial clades (the genera *Bacteroides* and *Prevotella*, and the families *Ruminococcaceae* and *Enterobacteriaceae*). (C) Fraction correlating (*q*≤0.1) to classes of Proteobacteria. (*** *p*≤10^−8^ by chi-squared test after Bonferroni correction; ** *p*≤10^−4^.) (D-E) Significant enrichment for variable gene families is not explained by taxon abundance or prevalence. log10(abundance) (D) and log10(prevalence) (E) were plotted vs. the degree of enrichment for variable gene families (a log-ratio of the number of significantly associated variable vs. invariable genes, with a pseudocount to avoid division by zero). Each family is represented as a circle; filled green circles represent significant (Bonferroni *p*<10^−2^) enrichments for variable, invariable, or non-significant gene families. Considering taxa with significant enrichments, there is no significant correlation with abundance (*r*=−0.1, *p*=0.58) or prevalence (*r*=−0.07, *p*=0.72). (PDF 200 kb)

Additional file 14 **Figure S16.** Proteobacteria, particularly *Enterobacteriaceae*, are still most strongly associated with variable gene families following clr-transformation. This transformation eliminates spurious correlation arising from the analysis of compositional data such as taxonomic relative abundances (see Additional file 9: Supplementary Information for details). (A–C) Associations of phylum abundances with gene families. Associations were computed as in Fig. 7 except using clr-transformed data, with an association significance threshold of (A) *q*≤0.05, (B) *q*≤0.035, and (C) *q*≤0.02. (D–F) Same as A–C, but for clr-transformed “enterotype” taxa (compare Figure S12B). (G) Same as A and D, but for clr-transformed taxonomic families. (H-I) Significant enrichment for variable/invariable gene families, based on clr-transformed data, plotted vs. (H) abundance and (I) prevalence (compare Figure S12D-E). (PDF 1177 kb)

Additional file 15 **Figure S13.** Genes only annotated in Proteobacteria or Euryarchaeota, but not Actinobacteria or Firmicutes, are more likely to be variable. (A) Bar plots give the fraction of gene families with at least one bacterial or archaeal representative in each category (significantly invariable, non-significant, and significantly variable) that were annotated *only*in the phylum listed (*x*-axis). Significance was assessed as in Additional file 13: Figure S12, using a Holm correction for significance. *p* values are color-coded by whether a phylum was enriched (*red*), depleted (*blue*), or neither (*gray*) for variable gene families (Holm-corrected *p*≤0.1). (B) Bar plots are as per (A), but test results come from a test sampling equal parts phylum-specific genes and genes annotated in all four listed phyla, with phylum-specific genes themselves uniformly sampled across phyla. (PDF 149 kb)

Additional file 16 **Figure S14.** Comparison between Bacteroidetes- and Firmicutes-specific variable and invariable genes. A-B) Venn diagrams showing the number of significantly variable (A) and invariable (B) gene families across Proteobacteria, Bacteroidetes, and Firmicutes, FDR ≤25*%*. Compare to Fig. 8 a, b. C) Bars indicate the fraction of phylum-specific variable gene families that were also variable overall (red, “both tests”) or that were specific to a particular phylum (yellow, “phylum-specific test only”). For the Bacteroidetes- (left) and Firmicutes- (right) specific tests, the proportion of invariable (blue), non-significant (gray), and variable (red) gene families, at an estimated 5% FDR (using cutoffs from overall test). Pathways with at least five total gene families across both phyla are shown. (D) Rectangular Venn diagrams showing the proportion of Bacteroidetes-specific (left), shared (center, bright), and Firmicutes-specific (right) invariable (blue) and variable (red) gene families for each of the pathways enumerated in A. (PDF 367 kb)

Additional file 17 Module and pathway enrichments for gene families with invariable abundances in every phylum-specific test (Fisher’s exact test, *q*≤0.25). (CSV 3 kb)

Additional file 18 Module and pathway enrichments for gene families variable in each phylum-specific test (Fisher’s exact test, *q*≤0.25). (CSV 2 kb)

Additional file 19 **Figure S15.** Distribution of proportions of zeros (i.e., proportion with read counts equal to zero) of invariable (FDR ≤0.05), non-significant (FDR ≤0.05), and variable (FDR ≤0.05) gene families identified by CCoDA. (PDF 138 kb)

Additional file 20 SRA IDs and characteristics (read length, average genome size from MicrobeCensus) for samples used in this study. (CSV 5 kb)

Additional file 21 **Figure S3.** Size parameter estimator choice affects accuracy of estimation. For each mock dataset *y*, simulated null data was generated from a negative binomial distribution, fixing the size parameter *k*_*y*_ but allowing the mean *μ*_*g*,*y*_ to vary for each of 1000 genes; simulated true-positive gene families were drawn from a negative binomial distribution with size equal to *z* *k*_*y*_ or *k*_*y*_/*z*, where *z* is the effect size. A-C) The choice of estimator affected the accuracy of size estimates. The mode method-of-moments estimator (C, *y*-axis) more accurately estimated the true size specified in the simulation (*x*-axis) than the harmonic mean (A, *y*-axis) or median (B, *y*-axis), and was more tolerant to differences in the ratio of true-positive variable and invariable gene families (colors). D-E) When the size parameter was known, *α*(D) and power (E) were well controlled, with *α* approximately equal to 0.05 at *p*≤0.05 and power approaching 1. Here, each simulation comprised three mock studies with different size parameters, mirroring our actual data. Bar heights represent means from four simulations and error bars are ±2 SD. The proportion of variable/invariable gene families was 0.5, and 44% of genes were true positives.(PDF 170 kb)

Additional file 22 **Figure S11.** Number of leaves correlates with tree density, but tree density corrects for the overall rate of evolution. The number of leaves (i.e., individual sequences) was plotted vs. tree density on a log-log scatter plot, with each circle representing one gene family. Two outliers with lower density than expected were plotted in colors: a putative transposase (green) and a *Staphylococcus* leukotoxin (red). Both families have large numbers of sequences from the same organism. (PDF 492 kb)

Additional file 23 Predicted tree densities. (CSV 314 kb)

Additional file 24 Source data for figures. Figure 1, source data 1: matrix of read counts (after rarefaction) for every gene family in each sample included in the present study. Figure 1, source data 2: matrix of average family lengths for every gene family in each sample included in the present study. Figure 1, source data 3: log-RPKG abundances for every gene family mapped in the present study. Figure 2, source data 1: residual log-RPKG abundances (i.e., after fitting the linear model) for every gene family mapped in the present study. Figure 3, source data 1: counts of invariable, non-significant, and variable gene families per pathway. “Strong,” “medium,” and “weak” refer to FDR cutoffs of 0.05, 0.10, and 0.25, respectively. Figure 3, source data 2: counts of invariable, non-significant, and variable gene families for ribosomes in each domain of life. Figure 4, source data 1: residual log-RPKG scaled by the expected variance under the null model (see the “Methods” section). Figure 6, source data 1: log_10_ phylogenetic distribution (PD), log_10_ residual variance statistics (residvar), significance at 5% FDR (invariable coded as “dn”, variable coded as “up”, non-significant coded as “ns”), presence in at least one bacterial/archaeal genome in KEGG, and annotations for all measured gene families. Figure 6, source data 2: counts of significant associations of invariable, non-significant, and variable gene families with taxonomic summary statistics. Figure 7, source data 1: counts of significant associations of invariable, non-significant, and variable gene families with phylum-level abundances. Figure 8, source data 1: *q* values for gene families in the overall test. Figure 8, source data 2: *q* values for gene families in phylum-specific tests. Figure 8, source data 3: JSON-formatted lists of significantly (in)variable or non-significant gene families at 5% (“strong”), 10% (“med”), and 25% FDR (“weak”); overall test. Figure 8, source data 4: JSON-formatted lists of significantly (in)variable or non-significant gene families at 5% (“strong”), 10% (“med”), and 25% FDR (“weak”); phylum-specific tests. (BZ 51464 kb)

## Abbreviations

AFL: Average family length
COG: Cluster of orthologous groups
FDR: False discovery rate
GC: Glucose control
GWAS: Genome-wide association study
GMH: Glyceromannoheptose
HMP: Human microbiome project
KEGG: Kyoto Encyclopedia of Genes and Genomes
Kdo: Ketodeoxyoctonate
LPS: Lipopolysaccharide
MAPE: Mean absolute percentage error
NB: Negative binomial. PD: Phylogenetic distribution
RPKG: Reads per kilobase of genome equivalents
T2D: Type II diabetes
T3SS, T4SS, T6SS: Type III, IV, and VI secretion systems
