# Supplementary figures and images for: Proteobacteria explain significant functional variability in the human gut microbiome
Source: Microbiome. 2017 Mar 23;5:36. doi: 10.1186/s40168-017-0244-z (PMC5363007; doi:10.1186/s40168-017-0244-z)

Supplemental Figure S1

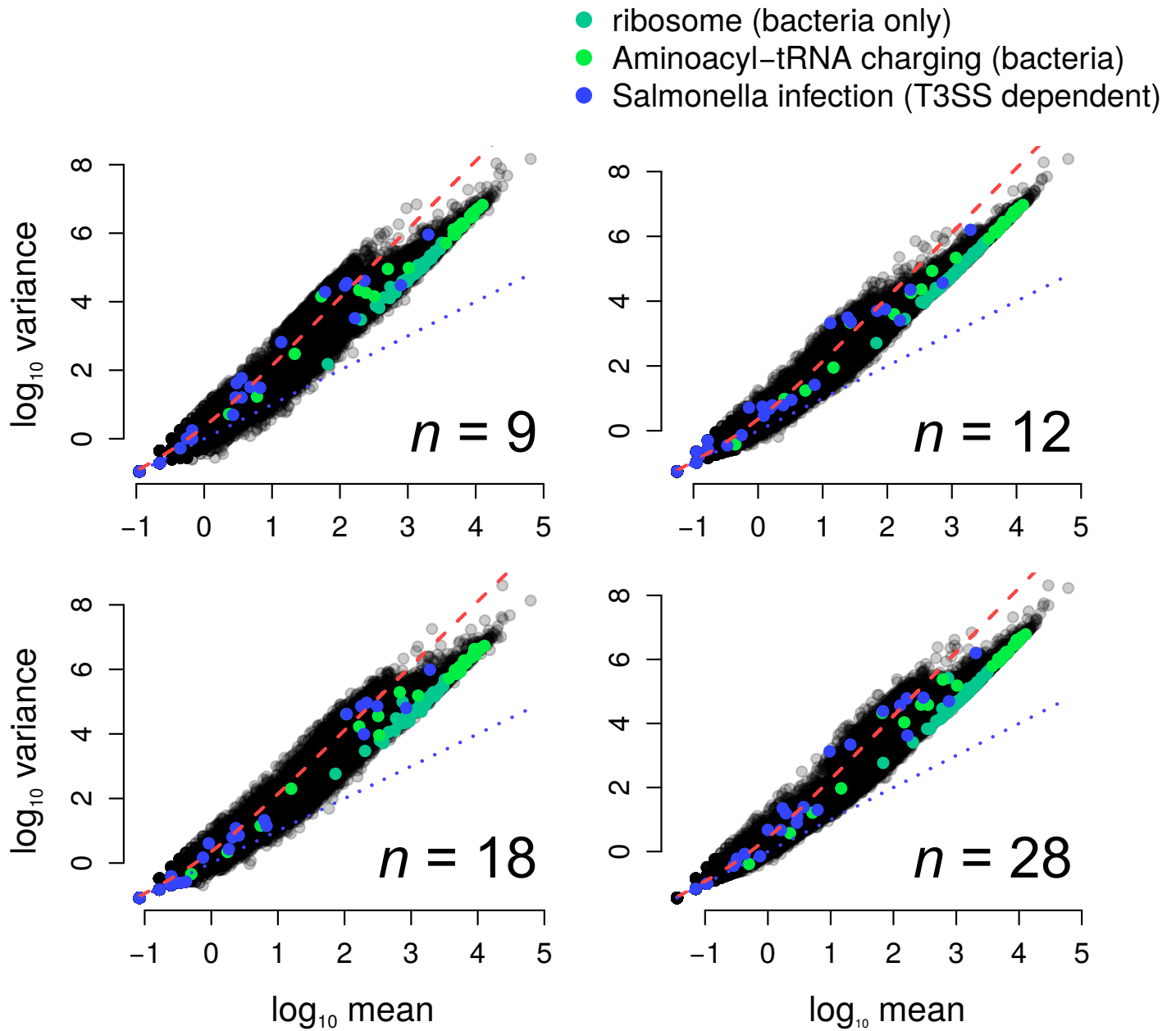

Supplement: Supplementary file 1 — Figure S1. The mean-variance relationship does not depend on the total number of samples. The glucose control (GC) study (n=37) was subsampled to various numbers of samples (9, 12, 18, 28), and the means, variances, and best-fits were computed as in Fig. 1, showing that this relationship is highly robust to sample size. (PDF 4298 kb) [file 40168_2017_244_MOESM1_ESM.pdf]

**A**

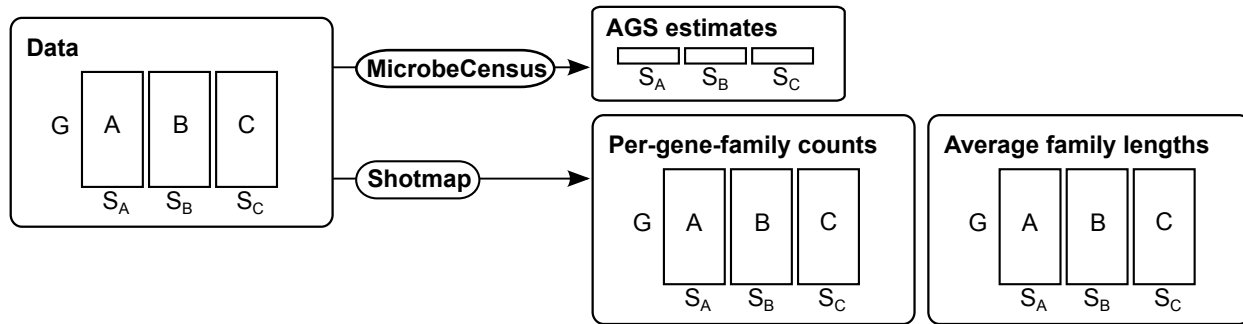

**B**

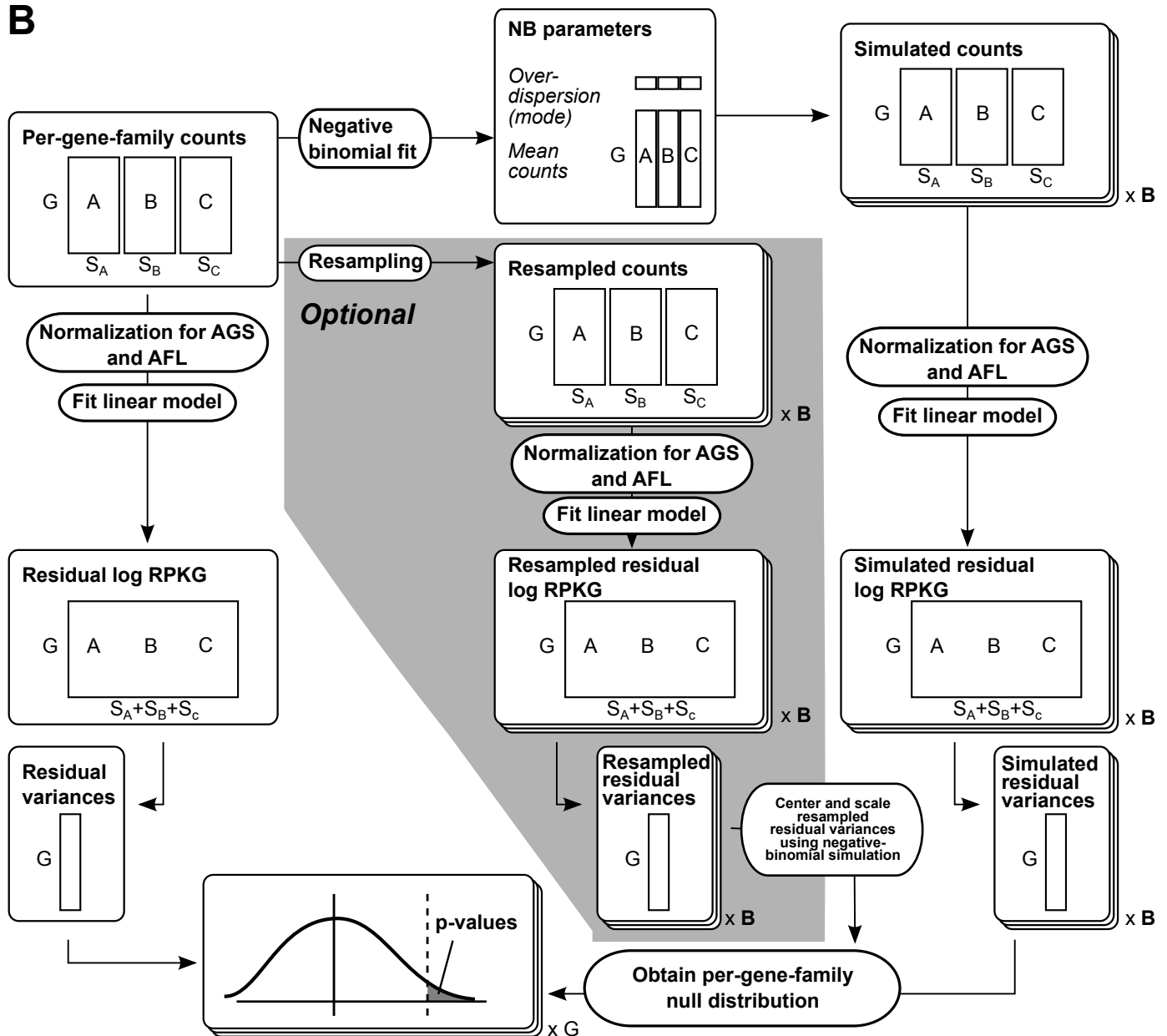

Supplement: Supplementary file 2 — Figure S2. Schematic shows overview of data processing and method. (A) Data were collected from multiple datasets, mapped using Shotmap [35] and normalized for average genome size [36] and average gene family length. (B) The test integrates multiple studies using a linear model, then uses a parametric bootstrap to generate the null distribution for this linear model’s residual variance. See Additional file 9 for a full description. (PDF 57 kb) [file 40168_2017_244_MOESM2_ESM.pdf]

Supplemental Figure S6

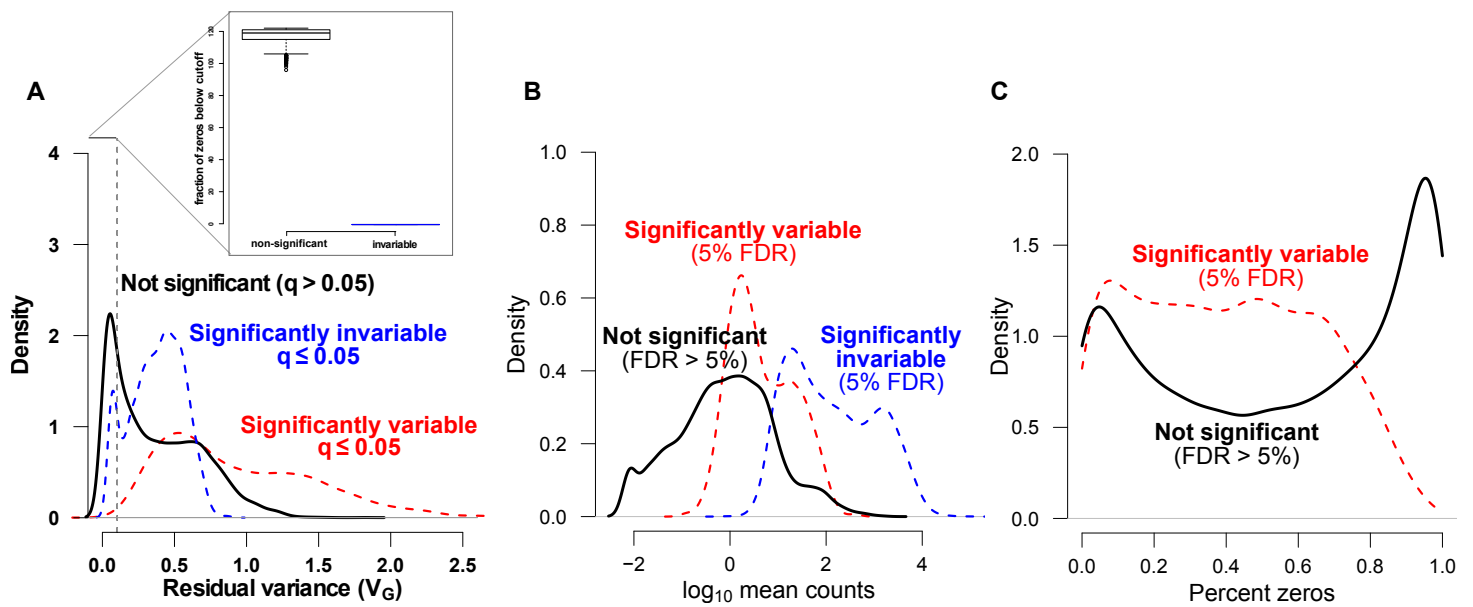

Supplement: Supplementary file 3 — Figure S6. We identified significantly variable and invariable gene families, which are not explained by means near the limit of detection or by large numbers of zeros. (A) Density plots of distributions of residual variance (V G) statistics for significantly invariable (blue dashed line), non-significant (black solid line), and significantly variable (red dashed line) gene families. The distributions had the expected trend (e.g., significantly variable gene families tended to have higher residual variance) but also overlapped, indicating the importance of the calculated null distribution. The inset shows the proportion of zero values for the non-significant (black) and significantly invariable (blue) gene families with V G falling in the lowest range (vertical dashed lines), indicating that the test differentiates between gene families that only appear invariable because they have few observations and gene families that are consistently abundant yet invariable. (B-C) Density plots of distributions of log10 mean counts (B) and fraction of zeros (C) across all three datasets for significantly invariable (blue dashed line), non-significant (black solid line), and significantly variable (red dashed line) gene families. Invariable gene families are not shown on the right because they overwhelmingly have small numbers of zeros. Gene families with very low mean abundances or large numbers of zeros tend to be called non-significant, not variable, indicating that the test correctly accounts for stochastic noise from low numbers of observations in determining statistical significance. (PDF 186 kb) [file 40168_2017_244_MOESM3_ESM.pdf]

Supplemental Figure S4

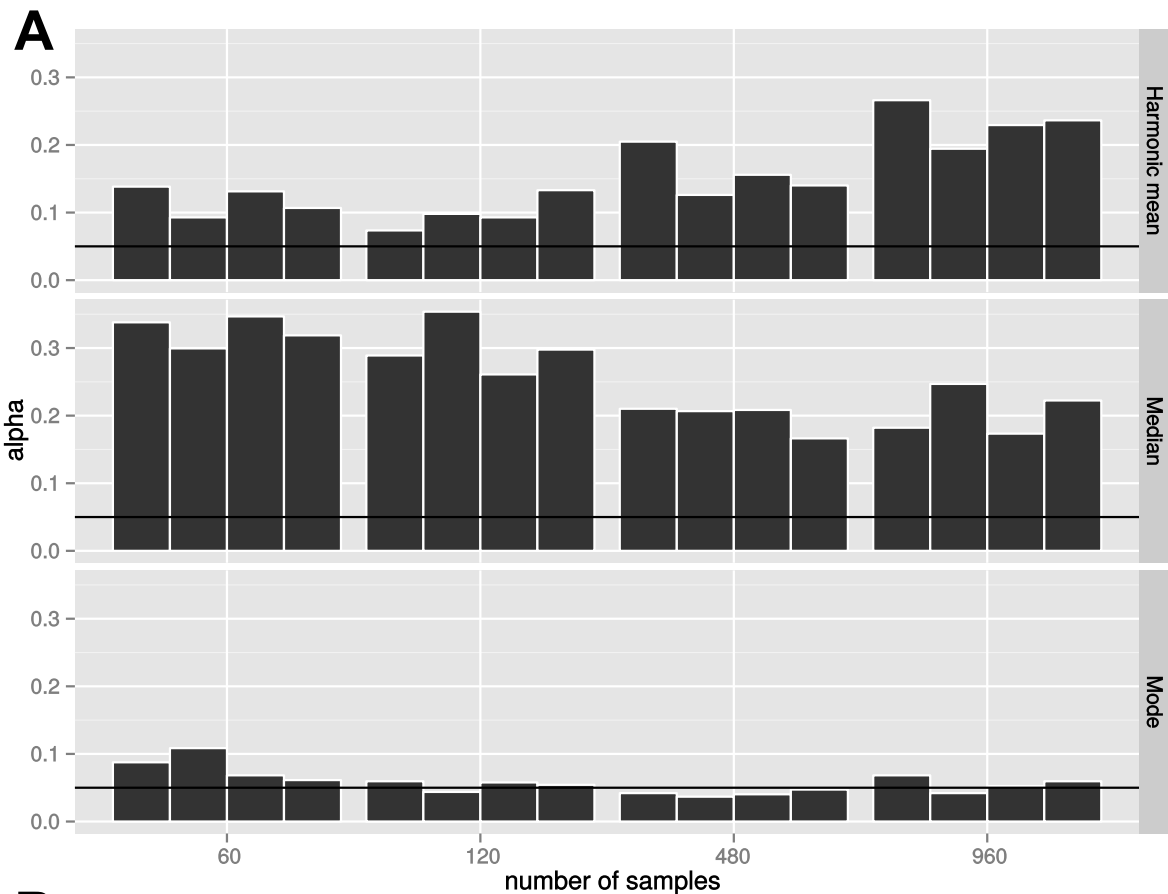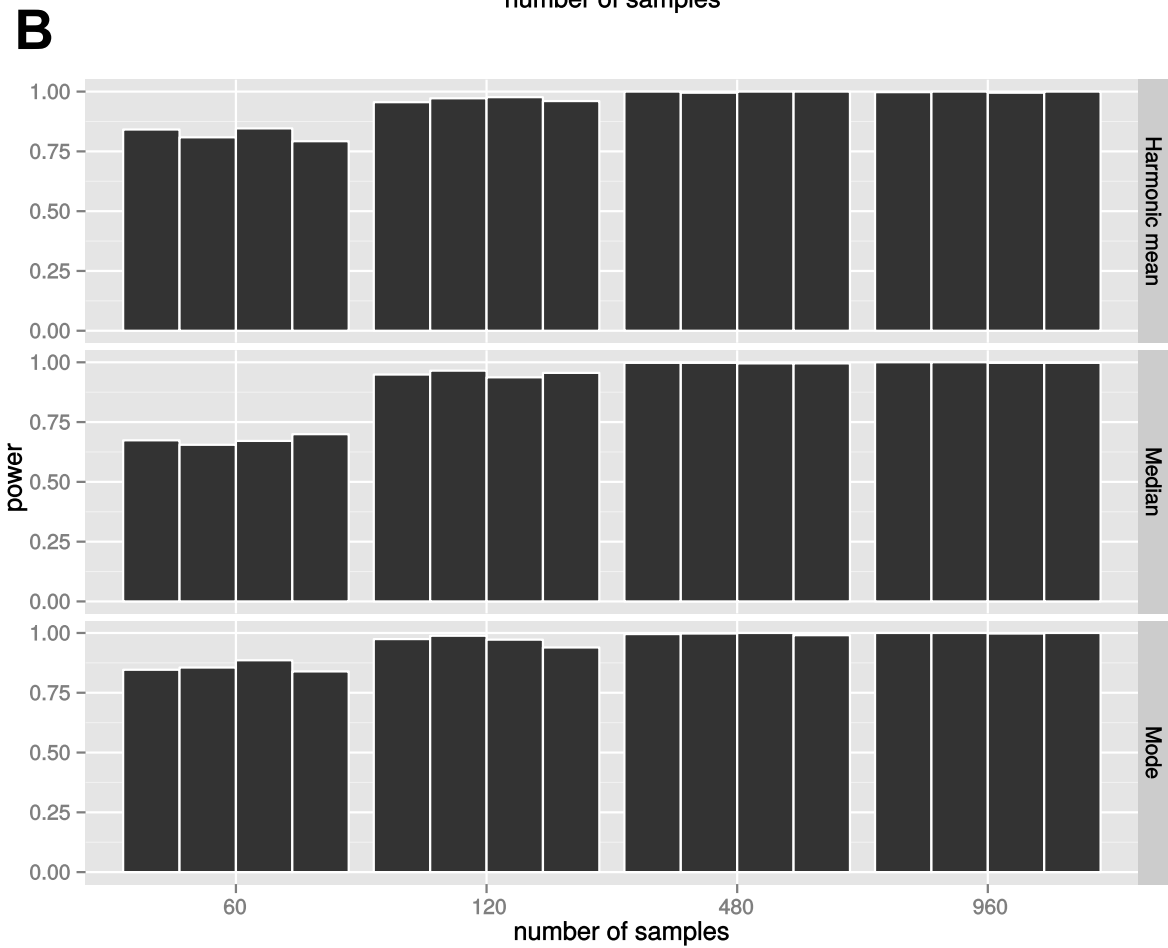

Supplement: Supplementary file 4 — Figure S4. Size parameter estimation affects power and α, with the mode method-of-moments giving the best control. α (A) was minimized and power (B) was maximized when the mode method-of-moments estimator was used to get estimates of the study-specific dispersion parameters $\widehat {k_{y}}$ky^. Bars are from four simulations. The proportion of variable/invariable gene families was 0.4, and 43% of genes were true positives. (PDF 44 kb) [file 40168_2017_244_MOESM4_ESM.pdf]

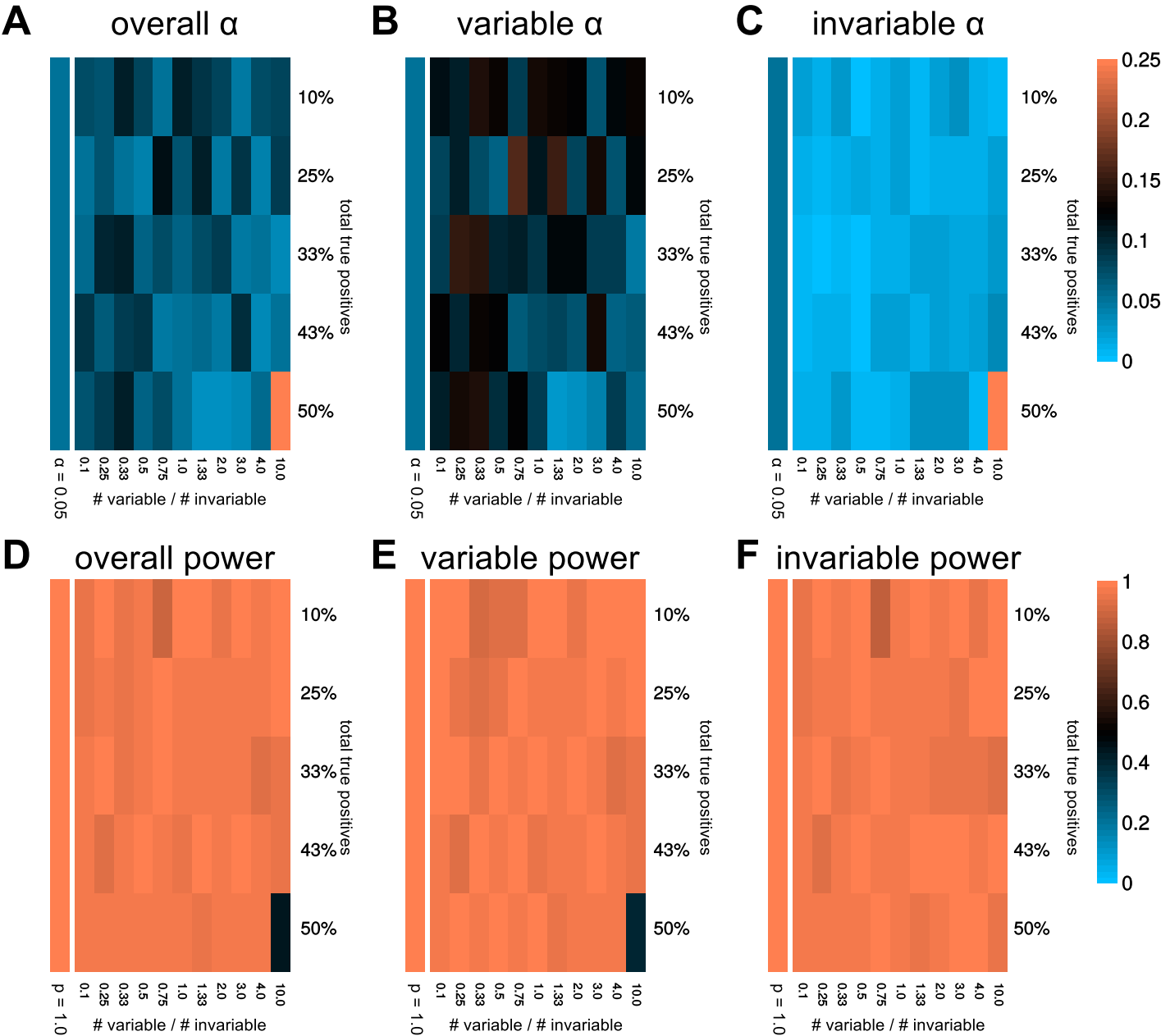

Supplement: Supplementary file 5 — Figure S5. The mode estimator is robust to changes in the proportion of true positives and the ratio of variable to invariable gene families. α (A-C) and power (D-F) as a function of the proportion of true positives (x-axis) and the ratio of variable to invariable true positives (y-axis) for n=120. α=0.05 and power =1 are shown in color-bars to the left of each heatmap for reference. α and power were calculated overall (left), for variable gene families (center), and for invariable gene families (right). In general, α was better controlled for the invariable gene families than for the variable gene families; we therefore used different empirical cutoffs for each set of genes. (PDF 131 kb) [file 40168_2017_244_MOESM5_ESM.pdf]

Supplemental Figure S7

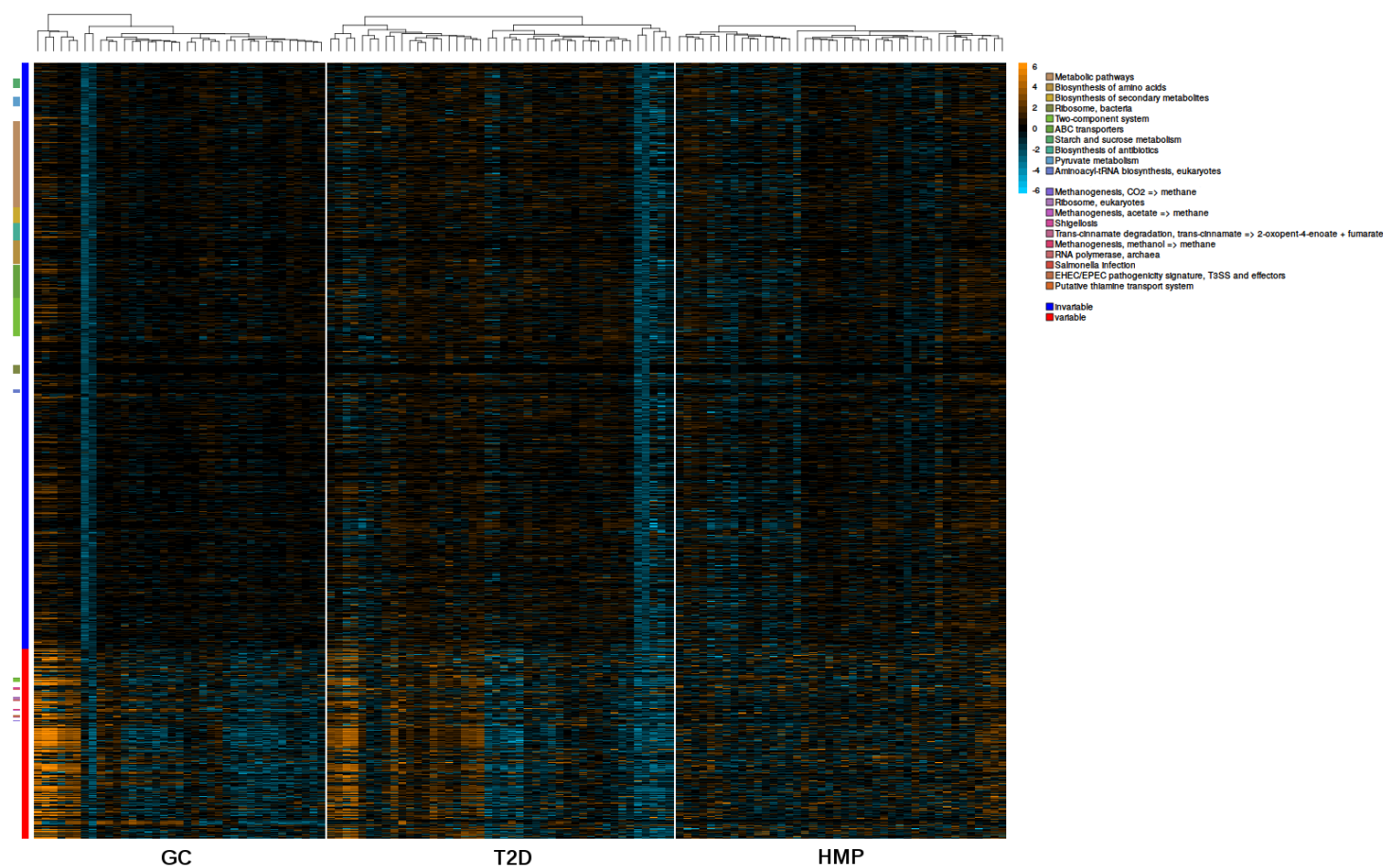

Supplement: Supplementary file 6 — Figure S7. Heatmap showing significantly variable and invariable gene families (unscaled). Heatmap showing residual log-RPKG abundances (i.e., after normalizing for between-study effects and gene-specific abundances) of significantly invariable (blue) and significantly variable (red) gene families. Variable and invariable gene families were clustered separately, while samples were clustered within each dataset. (PDF 158 kb) [file 40168_2017_244_MOESM6_ESM.pdf]

Supplemental Figure S8

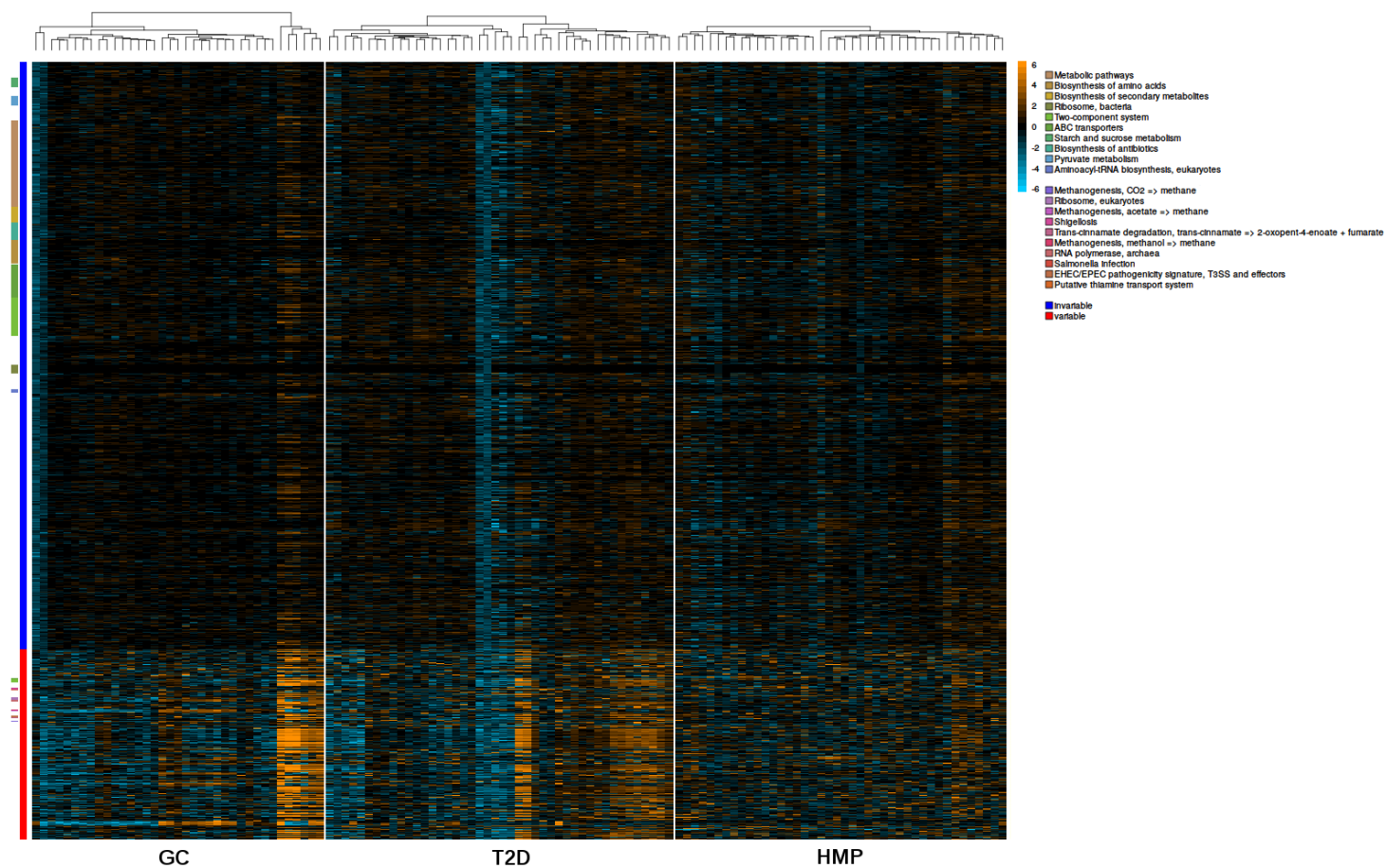

Supplement: Supplementary file 7 — Figure S8. Heatmap showing significantly variable and invariable gene families (scaled). As with Additional file 6: Figure S7, but residual log-RPKG abundances were scaled by their expected variance under the negative binomial null model (see the “Methods” section). (PDF 161 kb) [file 40168_2017_244_MOESM7_ESM.pdf]

**A**

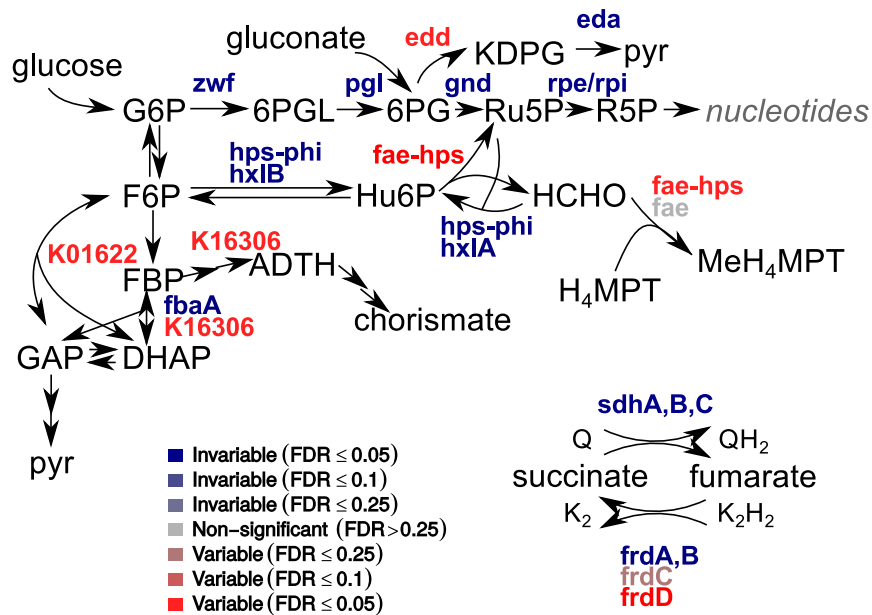

**B** GC T2D HMP

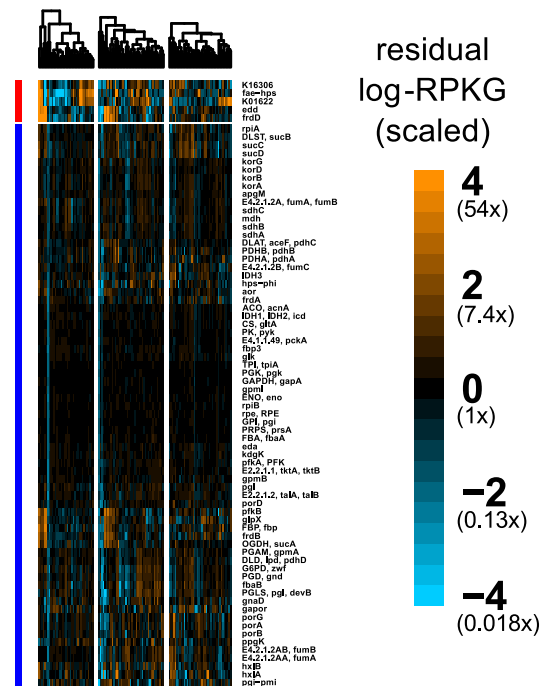

Supplement: Supplementary file 8 — Figure S9. Carbon metabolism contains variable and invariable gene families. (A) Pathway schematic showing a selection of measured gene families involved in central carbohydrate metabolism. Gene families are color-coded by whether they were variable (red) or invariable (blue), with strength of color corresponding to the FDR cutoff (color intensity). Genes involved in the Entner-Doudoroff pathway (edd), pentose metabolism (fae-hps), hexose metabolism (K01622, K16306), and tricarboxylic acid cycle intermediate metabolism (frdCD) were variable across healthy hosts. Abbreviated metabolites are glucose-6-phosphate (G6P), fructose-6-phosphate (F6P), fructose-1,6-bisphosphate (FBP), glyceraldehyde-3-phosphate (GAP), dihydroxyacetone phosphate (DHAP), 6-phosphogluconolactone (6PGL), 6-phosphogluconate (6PG), 2-keto-3-deoxy-phosphonogluconate (KDPG), ribulose-5-phosphate (R5P), ribose-5-phosphate (R5P), pyruvate (pyr), hexulose-6-phosphate (Hu6P), formaldehyde (HCHO), 2-amino-3,7-dideoxy-D-threo-hept-6-ulosonate (ADTH), and tetrahydromethanopterin (H4MPT). B) Heatmaps showing scaled residual log-RPKG for gene families (rows) involved in central carbohydrate metabolism. Variable (red) and invariable (blue) gene families were clustered separately, as were samples within a particular study (columns). log-RPKG values were scaled by the expected variance from the negative-binomial null distribution. (PDF 248 kb) [file 40168_2017_244_MOESM8_ESM.pdf]

Supplemental Figure S10

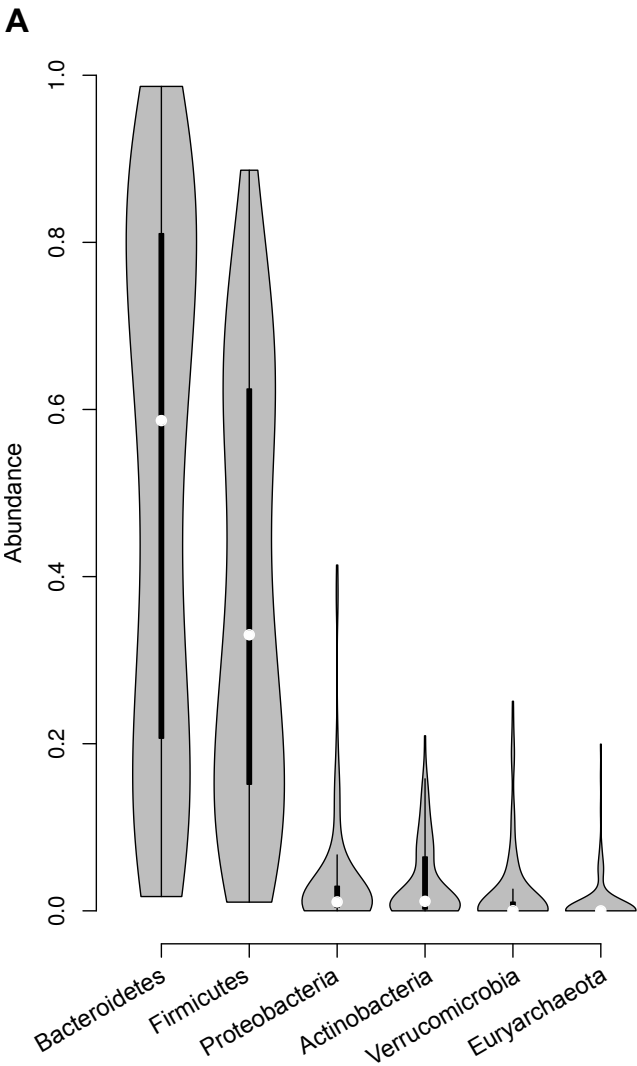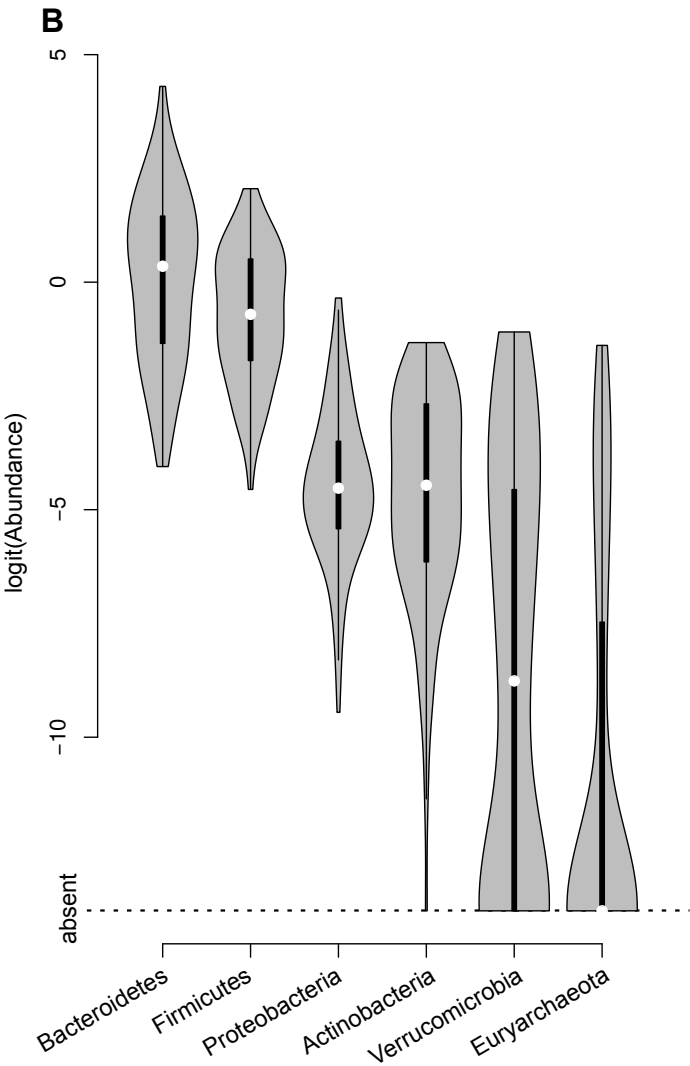

Supplement: Supplementary file 12 — Figure S10. Violin plots showing distributions of abundant phyla. (A) Abundance and (B) logit-transformed abundance ($\log {(\frac{a}{1-a}+10^{-6})}$log(a1−a+10−6), where 10−6 was added to prevent taking the log of zero) distributions were plotted for the six most abundant phyla. (PDF 155 kb) [file 40168_2017_244_MOESM12_ESM.pdf]

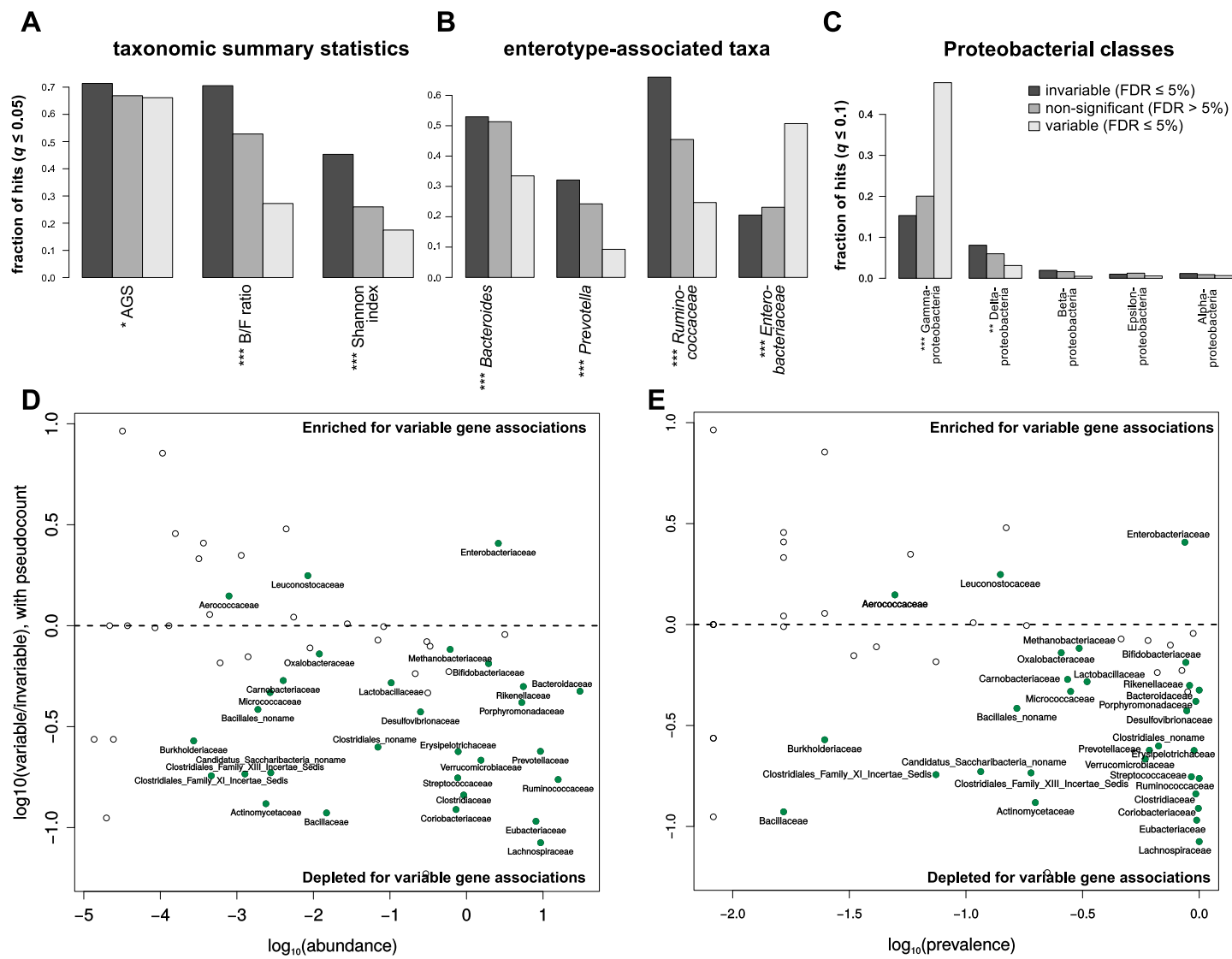

Supplement: Supplementary file 13 — Figure S12. Variable gene families are less-often correlated to measured host characteristics or enterotype-associated taxa and are more often correlated to Proteobacterial clades. (A-C) Bar plots give the fraction of gene families with at least one bacterial or archaeal representative in each category (significantly invariable, non-significant, and significantly variable) that were significantly correlated to various sample characteristics or taxonomic abundances, using partial Kendall’s τ to account for study effects and a permutation test to assess significance. (A) Fraction correlating (q≤0.05) to average genome size (AGS), the ratio of Bacteroidetes to Firmicutes (B/F ratio), and a measure of α-diversity (Shannon index). (B) Fraction correlating (q≤0.05) to the predicted abundance of specific bacterial clades (the genera Bacteroides and Prevotella, and the families Ruminococcaceae and Enterobacteriaceae). (C) Fraction correlating (q≤0.1) to classes of Proteobacteria. (*** p≤10−8 by chi-squared test after Bonferroni correction; ** p≤10−4.) (D-E) Significant enrichment for variable gene families is not explained by taxon abundance or prevalence. log10(abundance) (D) and log10(prevalence) (E) were plotted vs. the degree of enrichment for variable gene families (a log-ratio of the number of significantly associated variable vs. invariable genes, with a pseudocount to avoid division by zero). Each family is represented as a circle; filled green circles represent significant (Bonferroni p<10−2) enrichments for variable, invariable, or non-significant gene families. Considering taxa with significant enrichments, there is no significant correlation with abundance (r=−0.1, p=0.58) or prevalence (r=−0.07, p=0.72). (PDF 200 kb) [file 40168_2017_244_MOESM13_ESM.pdf]

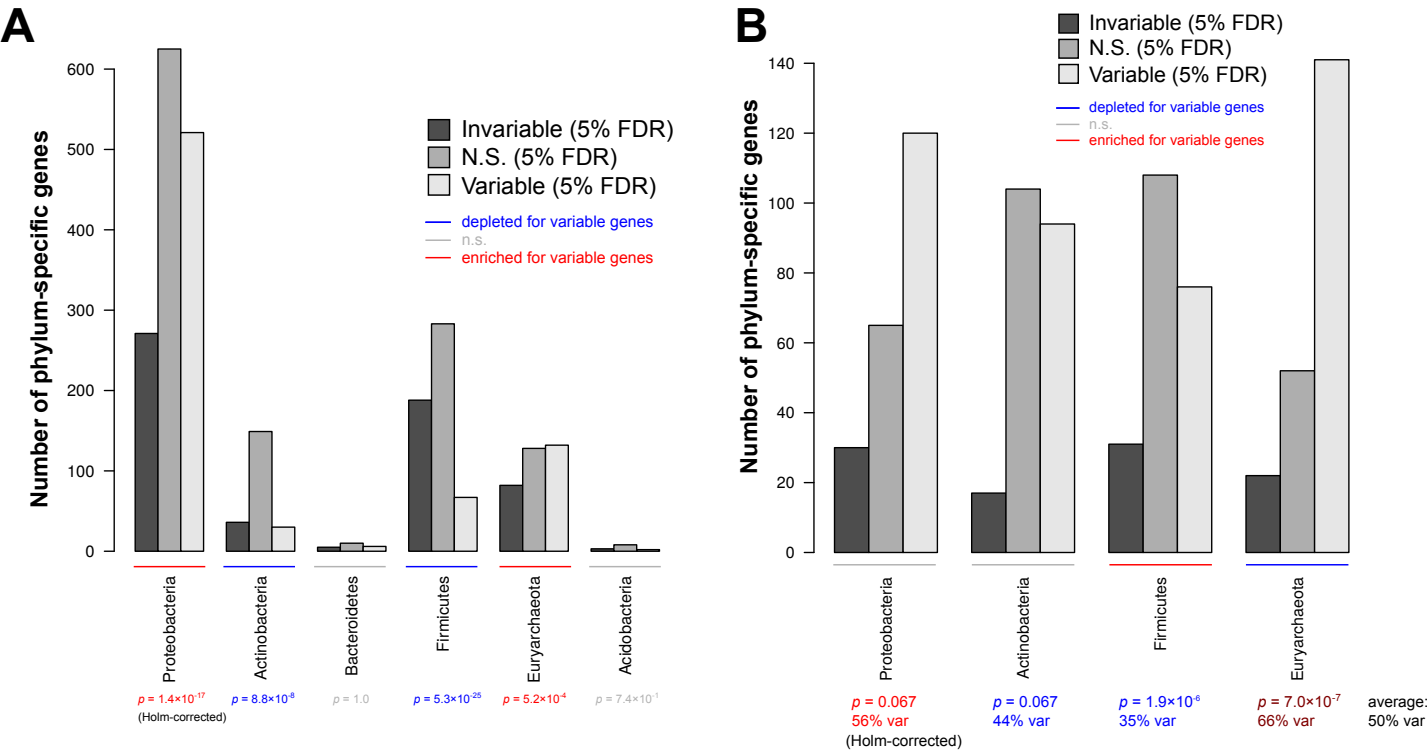

Supplement: Supplementary file 15 — Figure S13. Genes only annotated in Proteobacteria or Euryarchaeota, but not Actinobacteria or Firmicutes, are more likely to be variable. (A) Bar plots give the fraction of gene families with at least one bacterial or archaeal representative in each category (significantly invariable, non-significant, and significantly variable) that were annotated onlyin the phylum listed (x-axis). Significance was assessed as in Additional file 13: Figure S12, using a Holm correction for significance. p values are color-coded by whether a phylum was enriched (red), depleted (blue), or neither (gray) for variable gene families (Holm-corrected p≤0.1). (B) Bar plots are as per (A), but test results come from a test sampling equal parts phylum-specific genes and genes annotated in all four listed phyla, with phylum-specific genes themselves uniformly sampled across phyla. (PDF 149 kb) [file 40168_2017_244_MOESM15_ESM.pdf]

Supplemental Figure S14

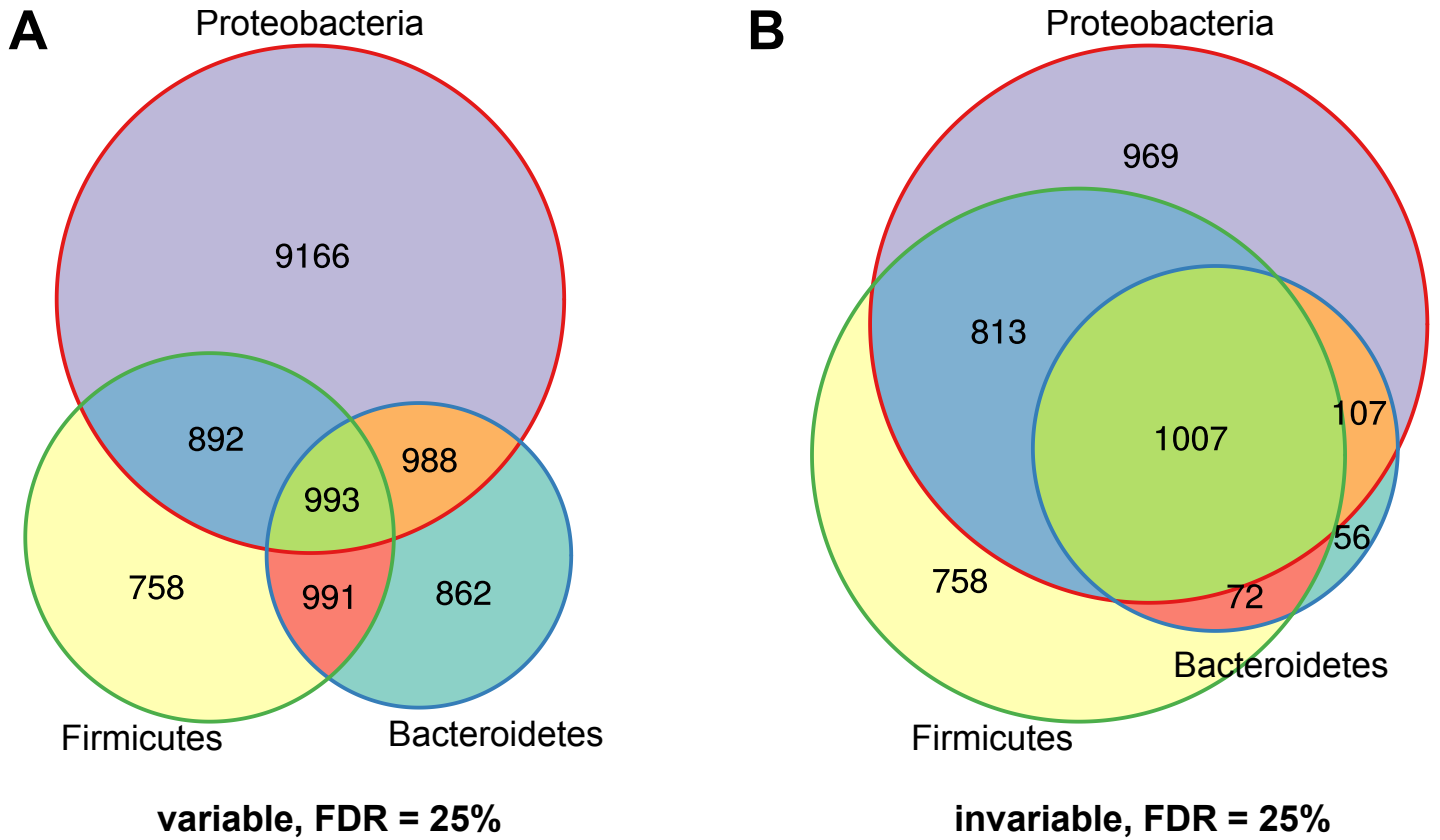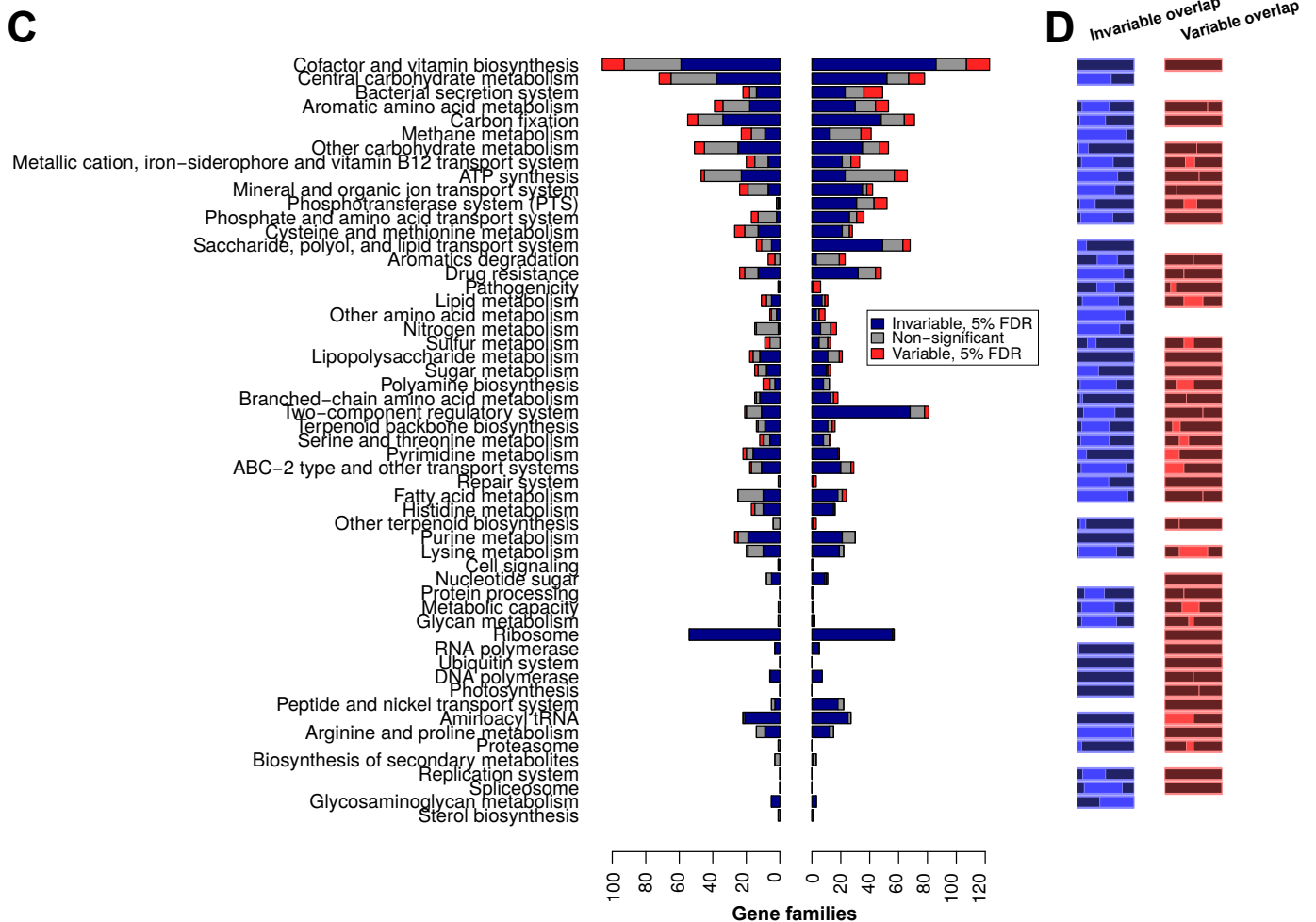

Supplement: Supplementary file 16 — Figure S14. Comparison between Bacteroidetes- and Firmicutes-specific variable and invariable genes. A-B) Venn diagrams showing the number of significantly variable (A) and invariable (B) gene families across Proteobacteria, Bacteroidetes, and Firmicutes, FDR ≤25%. Compare to Fig. 8 a, b. C) Bars indicate the fraction of phylum-specific variable gene families that were also variable overall (red, “both tests”) or that were specific to a particular phylum (yellow, “phylum-specific test only”). For the Bacteroidetes- (left) and Firmicutes- (right) specific tests, the proportion of invariable (blue), non-significant (gray), and variable (red) gene families, at an estimated 5% FDR (using cutoffs from overall test). Pathways with at least five total gene families across both phyla are shown. (D) Rectangular Venn diagrams showing the proportion of Bacteroidetes-specific (left), shared (center, bright), and Firmicutes-specific (right) invariable (blue) and variable (red) gene families for each of the pathways enumerated in A. (PDF 367 kb) [file 40168_2017_244_MOESM16_ESM.pdf]

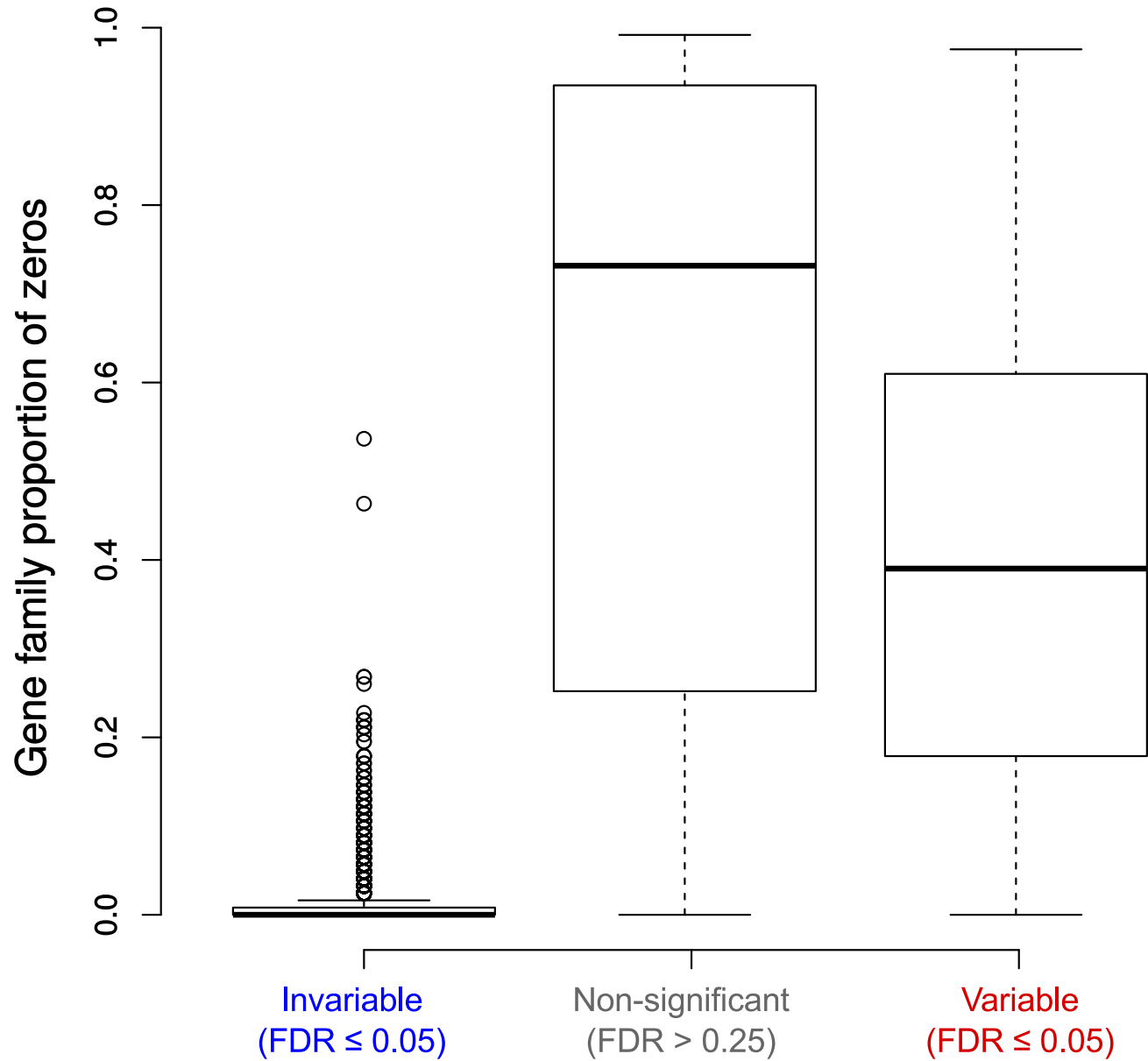

Supplement: Supplementary file 19 — Figure S15. Distribution of proportions of zeros (i.e., proportion with read counts equal to zero) of invariable (FDR ≤0.05), non-significant (FDR ≤0.05), and variable (FDR ≤0.05) gene families identified by CCoDA. (PDF 138 kb) [file 40168_2017_244_MOESM19_ESM.pdf]

Supplemental Figure S3

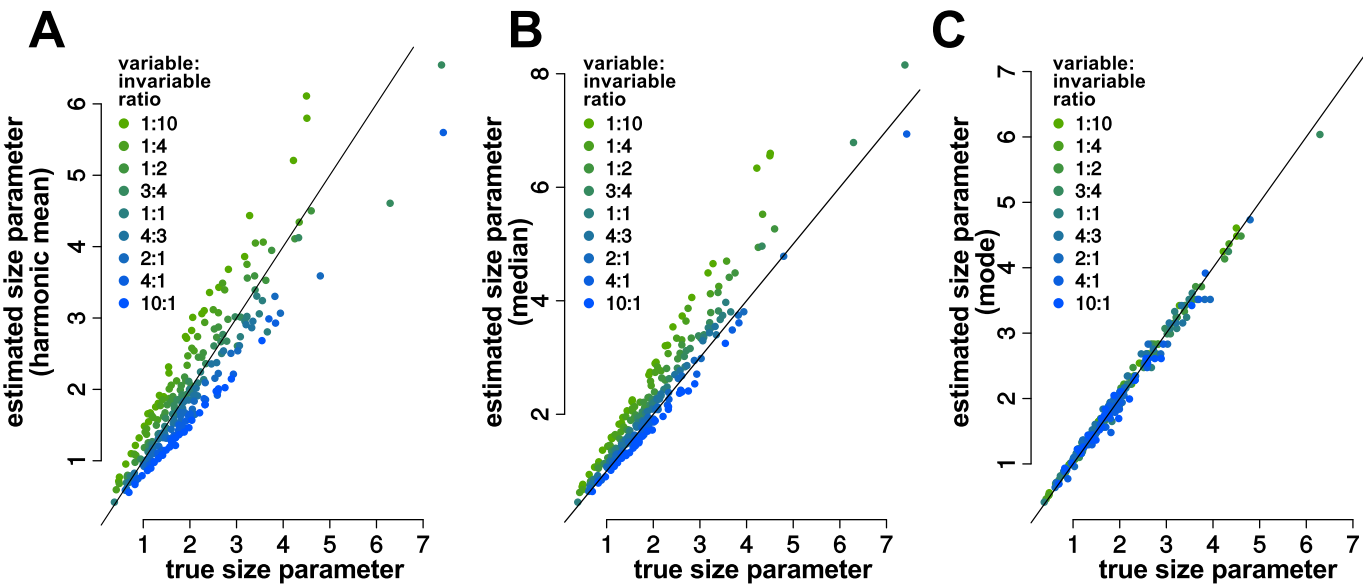

Size parameter known

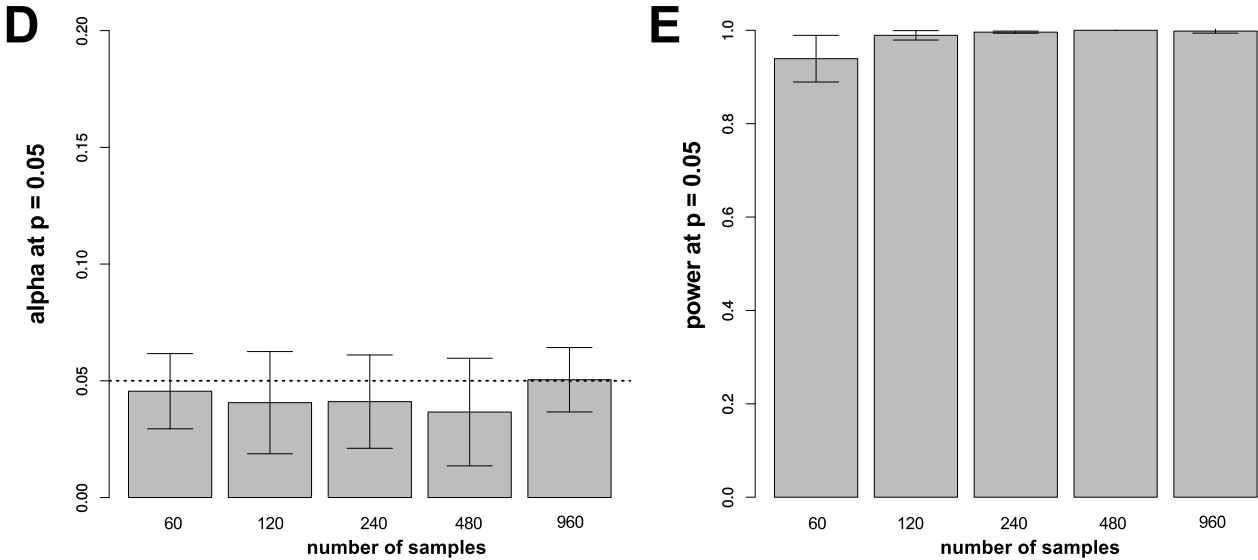

Supplement: Supplementary file 21 — Figure S3. Size parameter estimator choice affects accuracy of estimation. For each mock dataset y, simulated null data was generated from a negative binomial distribution, fixing the size parameter k y but allowing the mean μ g,y to vary for each of 1000 genes; simulated true-positive gene families were drawn from a negative binomial distribution with size equal to z k y or k y/z, where z is the effect size. A-C) The choice of estimator affected the accuracy of size estimates. The mode method-of-moments estimator (C, y-axis) more accurately estimated the true size specified in the simulation (x-axis) than the harmonic mean (A, y-axis) or median (B, y-axis), and was more tolerant to differences in the ratio of true-positive variable and invariable gene families (colors). D-E) When the size parameter was known, α(D) and power (E) were well controlled, with α approximately equal to 0.05 at p≤0.05 and power approaching 1. Here, each simulation comprised three mock studies with different size parameters, mirroring our actual data. Bar heights represent means from four simulations and error bars are ±2 SD. The proportion of variable/invariable gene families was 0.5, and 44% of genes were true positives.(PDF 170 kb) [file 40168_2017_244_MOESM21_ESM.pdf]

Supplemental Figure S11

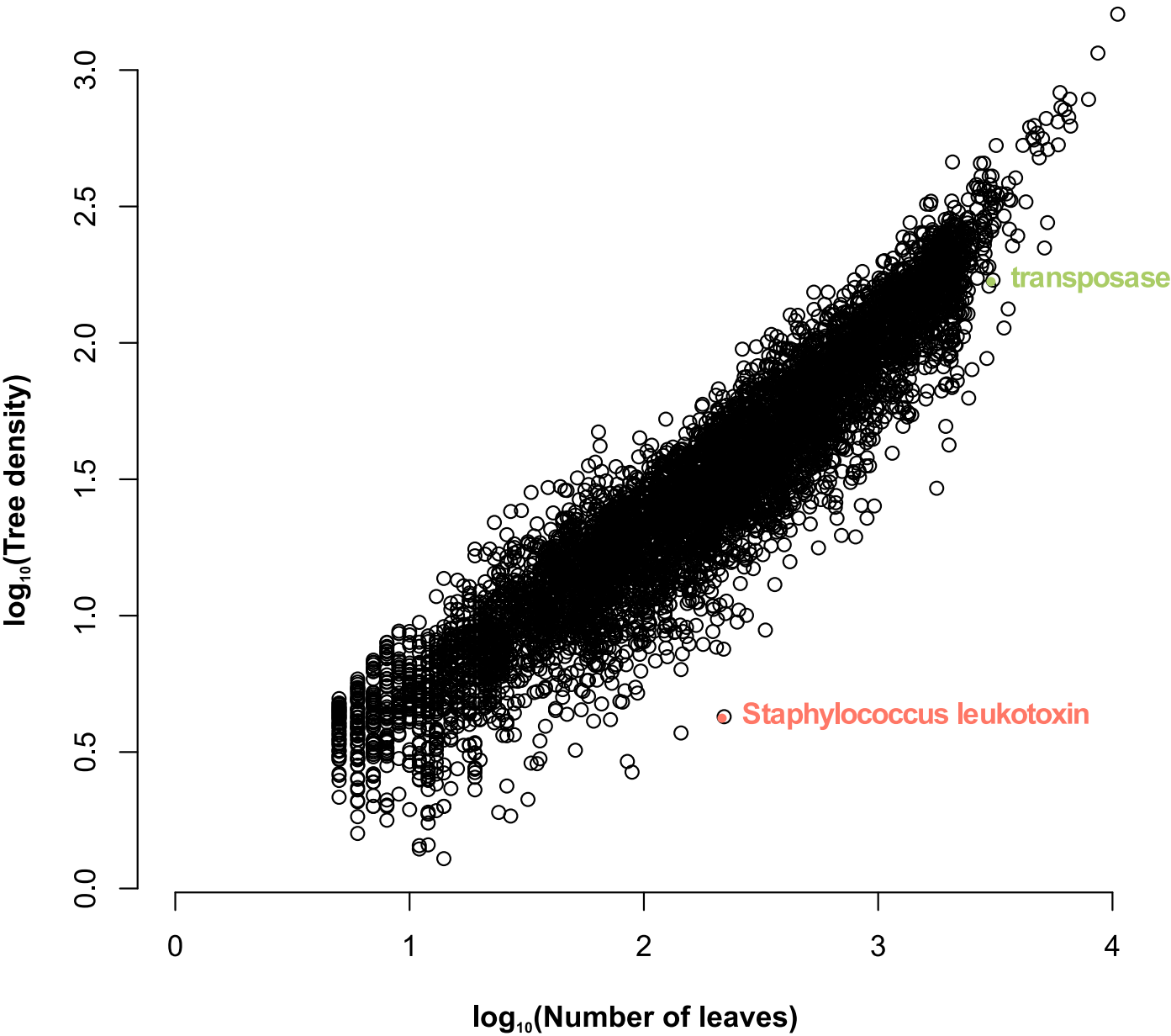

Supplement: Supplementary file 22 — Figure S11. Number of leaves correlates with tree density, but tree density corrects for the overall rate of evolution. The number of leaves (i.e., individual sequences) was plotted vs. tree density on a log-log scatter plot, with each circle representing one gene family. Two outliers with lower density than expected were plotted in colors: a putative transposase (green) and a Staphylococcus leukotoxin (red). Both families have large numbers of sequences from the same organism. (PDF 492 kb) [file 40168_2017_244_MOESM22_ESM.pdf]
