# Supplementary material for: Proteobacteria explain significant functional variability in the human gut microbiome
Source: Microbiome. 2017 Mar 23;5:36. doi: 10.1186/s40168-017-0244-z (PMC5363007; doi:10.1186/s40168-017-0244-z)
Supplement: Supplementary file 9 — Supplementary information. (PDF 98 kb) [file 40168_2017_244_MOESM9_ESM.pdf]

Supplemental Information

February 23, 2017

1 Description of schematic figure (Additional file 2: Figure S2)

(A) Data were processed by taking reads from multiple datasets (represented by letters here) with a certain number of samples (represented by S_A, S_B, etc.). These reads will eventually map to multiple gene families G. MicrobeCensus [1] was used to estimate average genome size, while Shotmap [2] was used to map reads, yielding both matrices of counts (right hand side) and matrices of average lengths of the best-hit proteins ("average family length" or AFL). AFL and AGS estimates were used to normalize counts.

(B) We calculate our statistic and assign p-values as follows. First, we normalized counts from Shotmap with AFL and AGS, log-transform the resulting reads per kilobase of genome (RPKG), then applied a simple linear model to fit dataset- and gene-family-specific effects. The resulting residuals ("residual log RPKG") formed a matrix of G genes by S_A+S_B+S_C samples. We took the variance across all samples for each gene to obtain a 1xG vector of residual variances. To get a null distribution, we could either use data generated from the negative binomial fit (parametric bootstrapping) or, optionally, from a negative binomial fit integrated with (shaded section) non-parametric bootstrap resampling. For the negative binomial fit, from the count matrices, we estimated the mean of each gene in each dataset, as well as dataset-specific overdispersion parameters k. We then used these to make simulated count datasets ("$\times$ B" indicating that this card is replicated once for each of B simulations), which we process as in the case of the real data, yielding simulated log-RPKG matrices and simulated residual variances for each gene family. For the resampling (if applicable), we sampled with replacement from each count dataset, yielding resampled counts. We processed these in the same way to obtain resampled residual variances. Finally, if using the resampled data, we centered and scaled the resampled residual variances using per-gene-family means and standard deviations from the simulated residual variances; otherwise, we simply took the values from applying the test to the negative binomial simulations. These formed the background distribution (bottom panel, solid curve) for each gene in G ("$\times$ G" indicating that this card is replicated once for each of G genes). The actual observed residual variance (dashed line) was then compared to this distribution to obtain p-values (gray shaded area).

2 Carbon metabolism contains variable gene families participating in alternative pathways

Glucose can be metabolized by two alternative pathways: the well-known Embden-Meyerhof-Parnas (EMP) pathway (i.e., classical "glycolysis"), or the Entner-Doudoroff pathway (ED). Both take glucose to

pyruvate, but with differing yields of ATP and electron carriers; ED also allows growth on sugar acids like gluconate [3]. Our analysis indicates that hosts differ in how much their gut microbial communities use ED. While all genes in the "core module" of glycolysis dealing with 3-carbon compounds were significantly invariable across individuals, we found that the ED-specific gene family *edd*, which takes 6-phosphogluconate to 2-keto-3-deoxy-phosphogluconate (KDPG), was significantly variable.

We also discovered significant variability in other enzymes involved in unusual sugar-phosphate and tricarboxylic acid metabolism. Multifunctional and primarily archaeal variants of fructose-bisphosphate aldolase (K16306, K01622) were significantly variable across hosts, while the typical FBA enzyme (*fbaA*) was significantly invariable. Another difference was seen in genes potentially contributing to ribose-phosphate generation. While typical pentose-phosphate pathway genes (e.g., the glucose-6-phosphate 1-dehydrogenase *zwf* and the 6-phosphogluconate dehydrogenase *gnd*) were invariable, the bifunctional gene family *fae-hps*, thought to be involved in an alternative route to ribose-phosphate, was significantly variable [4]. Finally, a subunit of fumarate reductase, *frdD*, was also significantly variable. Fumarate reductase catalyzes the reverse reaction from the typical TCA cycle enzyme succinate dehydrogenase and can be used for redox balance during anaerobic growth [5]. Conversely, the standard succinate dehydrogenase genes *sdhA*, *sdhB* and *sdhC* were significantly invariable. These results suggest that using our test to identify variable genes within otherwise invariable pathways can reveal diverged functionality as well as families that play domain or clade-specific roles.

3 Note on LPS modifications

Hexaacylated lipid A, as found in *E. coli*, stimulates TLR4 and induces the release of pro-inflammatory cytokines; conversely, pentaacylated lipid A variants, as found in *Bacteroides*, tend not to induce TLR4 signaling, and can even prevent the hexaacylated variety from inducing inflammation [6]. This inflammation may have a variety of downstream effects on health. For example, elevated serum LPS levels are observed in obese individuals [7, 8] and individuals with inflammatory bowel disease [7], and have been linked to an increase in coronary heart disease events [9]. Conversely, a recent study advanced the hypothesis that dampening of TLR4 signaling in childhood by *Bacteroides* species may actually *increase* later susceptibility to autoimmune disease [6].

4 Correlation of variable and invariable gene families with taxonomic summary statistics

We examined correlations between gene abundance and three taxonomic summary statistics that have been previously linked to microbiome function: average genome size (AGS) [1], the Bacteroidetes/Firmicutes ratio [10, 11], and α-diversity (Shannon index, or $H_s = -\sum_i a_{i,s} \log a_{i,s}$, where a is the abundance of taxon i in sample s).

It has previously been suggested [1] that the genome size of gut microbiota reflects a trade-off between specialization (in which metabolic pathways for the production of reliably present nutrients may be lost over time, potentially resulting in auxotrophy) and generalization, or the ability to survive and grow in different metabolic conditions (which may require more biosynthetic genes). AGS itself has also been linked to health outcomes; for instance, individuals with Crohn's disease tend to have gut microbiota with

larger genome size [2]. However, variable gene families were no more likely to be associated with AGS. Only 66% of variable gene families (with at least one bacterial or archaeal representative) had abundances that were significantly correlated with average genome size ($q \leq 0.05$), compared to 71% of invariable gene families and 66% of non-significant families at the same threshold. Thus, genome size correlates generally with gene abundance but does not predict variability of genes in healthy hosts.

The most dominant phylum-level trend across healthy human gut microbiomes is the trade-off between the two dominant phyla, Bacteroidetes and Firmicutes. The ratio of these two phyla (B/F ratio) has been linked to obesity in some studies [10, 11]; however, a later meta-analysis [12] revealed no consistent correlation across studies. Here, we found that variable genes were actually substantially *less* likely to be correlated to the B/F ratio (27%, $q \leq 0.05$) than either invariable (71%) or non-significantly-associated (55%) genes. These results parallel what we observe when we correlate gene family abundances with the α-diversity of observed bacterial species. We estimated α-diversity using the Shannon index, which is low when the distribution of species abundance is highly skewed, and high when there are many species of even abundance. Only 17% of significantly variable genes correlate significantly to the Shannon diversity ($q \leq 0.05$), versus 45% of significantly invariable and 26% of non-significant genes. We therefore conclude that bacterial and archaeal gene families identified as variable in this study are less likely to be associated with average genome size, B/F ratio, or α-diversity.

When examining the PD-stratified gene families, we noticed that the variable/high-PD gene set was also enriched for gene families described as “hypothetical” in the KEGG Orthology database; hypothetical gene families were also observed in the invariable/low-PD set, but they were statistically depleted (see main text). We were interested in whether these conserved-yet-variable hypothetical gene families could be acting as markers for minor phyla. Indeed, out of 81 genes in this group, 44 were significantly associated with Proteobacterial abundance ($q \leq 0.05$ by the above Kendall’s partial τ test) and 13 were associated with Actinobacteria at the same threshold. However, 5 and 7 each were associated with Firmicutes and Bacteroidetes, indicating that even the major phyla of the human gut vary with respect to certain as-yet-uncharacterized functions.

5 Taxonomic associations with transformed compositional data are similar

Because relative abundances of taxa are compositional data (i.e., sum to 100% within a sample), we wanted to make sure that the associations we found between variable gene families and Proteobacteria were not artifacts of “spurious correlation” potentially caused by this constraint ([13, 14]). (The analyses in Additional file 13: Figure S13 are not affected by this issue since they are not based on correlation.) We therefore performed a variation of the centered log-ratio (clr) transform, a data transformation for compositional data first proposed by Aitchison [13].

Briefly, this transformation expresses each taxon’s relative abundance as a log-ratio of the geometric mean abundance of all taxa within that sample. Zero values are typically modified by a pseudocount. The transformation can be described according to the equation:

$$c_{t,s} = \log \frac{(a_{t,s} + p)/(1 + p)}{g(a_{x \in T,s})}$$

where c is the clr-transformed abundance, a is the original relative abundance, t is a particular taxon in the set of taxa measured T, s is a particular sample, p is a pseudocount, and $g(y)$ is the geometric mean of the quantity y. We used a pseudocount of half the minimum non-zero value in the abundance matrix: for phyla, this pseudocount was 5.5×10^{-5}. (Because many phyla detected in at least one sample were often absent and their abundances would therefore be driven by the pseudocount, we used only taxa present in at least 75% of samples to calculate the geometric mean in the denominator.) The transformation was applied separately to relative abundance matrices of phyla, families, and genera as calculated by MetaPhlAn2, as described in the main-text section "Methods". Finally, association tests between taxa and gene families were performed as described in the main text, section "Methods," under the heading "Associations with clinical and taxonomic variables."

The results were broadly similar to the results on non-transformed data (see Additional File 16, Figure S16). Proteobacteria remained the predominant source of variable gene families after phylum abundances were clr-transformed. Actinobacteria and Firmicutes contributed more variable gene families when transformed vs. non-transformed data were used, although these associations tended to be weaker than the associations for Proteobacteria and Firmicutes, as could be seen by making the FDR threshold for association more strict (Additional File 16, Figure S16A-C). Associations with enterotype taxa showed the same pattern using transformed (Figure S16D-F) vs. non-transformed data (Additional File 12, Figure S12B).

Finally, associations across all bacterial and archaeal families (Figure S16G) show that *Enterobacteriaceae*, a Proteobacterial family, remained the most associated with variable gene families, followed by *Bifidobacteriaceae*, an Actinobacterial family, and *Methanobacteriaceae*, a family of Archaea. After the *Enterobacteriaceae*, the *Micrococcaceae*, an Actinobacterial family, was the next-most-prevalent and -abundant family to be as strongly enriched for variable genes (Figure S16H-I); in the gut, representatives of this family detected by MetaPhlAn included only *Rothia mucilaginosa* and unclassified *Rothia* species. Unlike *Enterobacteriaceae*, *Bifidobacteriaceae*, and *Methanobacteriaceae*, *Rothia mucilaginosa* has mainly been described in the literature as a commensal member of the oropharyngeal flora and as an opportunistic pulmonary pathogen [15]. Overall, while Proteobacterial clades continue to be the most-associated with healthy gut functional variability after clr-transformation, all of these taxa represent intriguing targets for further study.

References

[1] Nayfach S, Pollard KS. Average genome size estimation improves comparative metagenomics and sheds light on the functional ecology of the human microbiome. Genome Biol. 2015 Jan;16:51.

[2] Nayfach S, Bradley PH, Wyman SK, Laurent TJ, Williams A, Eisen JA, et al. Automated and accurate estimation of gene family abundance from shotgun metagenomes. PLoS Comput Biol. 2015 Nov;11(11):e1004573.

[3] Peekhaus N, Conway T. What's for dinner?: Entner-Doudoroff metabolism in *Escherichia coli*. J Bacteriol. 1998 Jul;180(14):3495–502.

[4] Goenrich M, Thauer RK, Yurimoto H, Kato N. Formaldehyde activating enzyme (Fae) and hexulose-6-phosphate synthase (Hps) in *Methanosarcina barkeri*: a possible function in ribose-5-phosphate biosynthesis. Arch Microbiol. 2005 Oct;184(1):41–48.

[5] Spencer ME, Guest JR. Isolation and properties of fumarate reductase mutants of *Escherichia coli.* J Bacteriol. 1973 May;114(2):563–70.

[6] Vatanen T, Kostic AD, D'Hennezel E, Siljander H, Franzosa EA, Yassour M, et al. Variation in microbiome LPS immunogenicity contributes to autoimmunity in humans. Cell. 2016;165(4):842–853.

[7] Gardiner KR, Halliday MI, Barclay GR, Milne L, Brown D, Stephens S, et al. Significance of systemic endotoxaemia in inflammatory bowel disease. Gut. 1995;36(6):897–901.

[8] Boutagy NE, McMillan RP, Frisard MI, Hulver MW. Metabolic endotoxemia with obesity: Is it real and is it relevant? Biochimie. 2016;124:11–20.

[9] Kallio KAE, Hätönen KA, Lehto M, Salomaa V, Männistö S, Pussinen PJ. Endotoxemia, nutrition, and cardiometabolic disorders. Acta Diabetol. 2015 Apr;52(2):395–404.

[10] Turnbaugh PJ, Hamady M, Yatsunenko T, Cantarel BL, Duncan A, Ley RE, et al. A core gut microbiome in obese and lean twins. Nature. 2009 Jan;457(7228):480–4.

[11] Ley RE, Bäckhed F, Turnbaugh P, Lozupone CA, Knight RD, Gordon JI. Obesity alters gut microbial ecology. Proc Natl Acad Sci U S A. 2005 Aug;102(31):11070–5.

[12] Finucane MM, Sharpton TJ, Laurent TJ, Pollard KS. A taxonomic signature of obesity in the microbiome? Getting to the guts of the matter. PLoS ONE. 2014 Jan;9(1):e84689.

[13] Aitchison J. The statistical analysis of compositional data. Chapman and Hall; 1986.

[14] Fernandes AD, Reid JN, Macklaim JM, McMurrough TA, Edgell DR, Gloor GB. Unifying the analysis of high-throughput sequencing datasets: characterizing RNA-seq, 16S rRNA gene sequencing and selective growth experiments by compositional data analysis. Microbiome. 2014;2(1):15.

[15] Maraki S, Papadakis IS. *Rothia mucilaginosa* pneumonia: a literature review. Infect Dis. 2015 Mar;47(3):125–129.
