# Supplementary material for: Proteobacteria explain significant functional variability in the human gut microbiome
Source: Microbiome. 2017 Mar 23;5:36. doi: 10.1186/s40168-017-0244-z (PMC5363007; doi:10.1186/s40168-017-0244-z)
Supplement: Supplementary file 14 — Figure S16. Proteobacteria, particularly Enterobacteriaceae, are still most strongly associated with variable gene families following clr-transformation. This transformation eliminates spurious correlation arising from the analysis of compositional data such as taxonomic relative abundances (see Additional file 9: Supplementary Information for details). (A–C) Associations of phylum abundances with gene families. Associations were computed as in Fig. 7 except using clr-transformed data, with an association significance threshold of (A) q≤0.05, (B) q≤0.035, and (C) q≤0.02. (D–F) Same as A–C, but for clr-transformed “enterotype” taxa (compare Figure S12B). (G) Same as A and D, but for clr-transformed taxonomic families. (H-I) Significant enrichment for variable/invariable gene families, based on clr-transformed data, plotted vs. (H) abundance and (I) prevalence (compare Figure S12D-E). (PDF 1177 kb) [file 40168_2017_244_MOESM14_ESM.pdf]

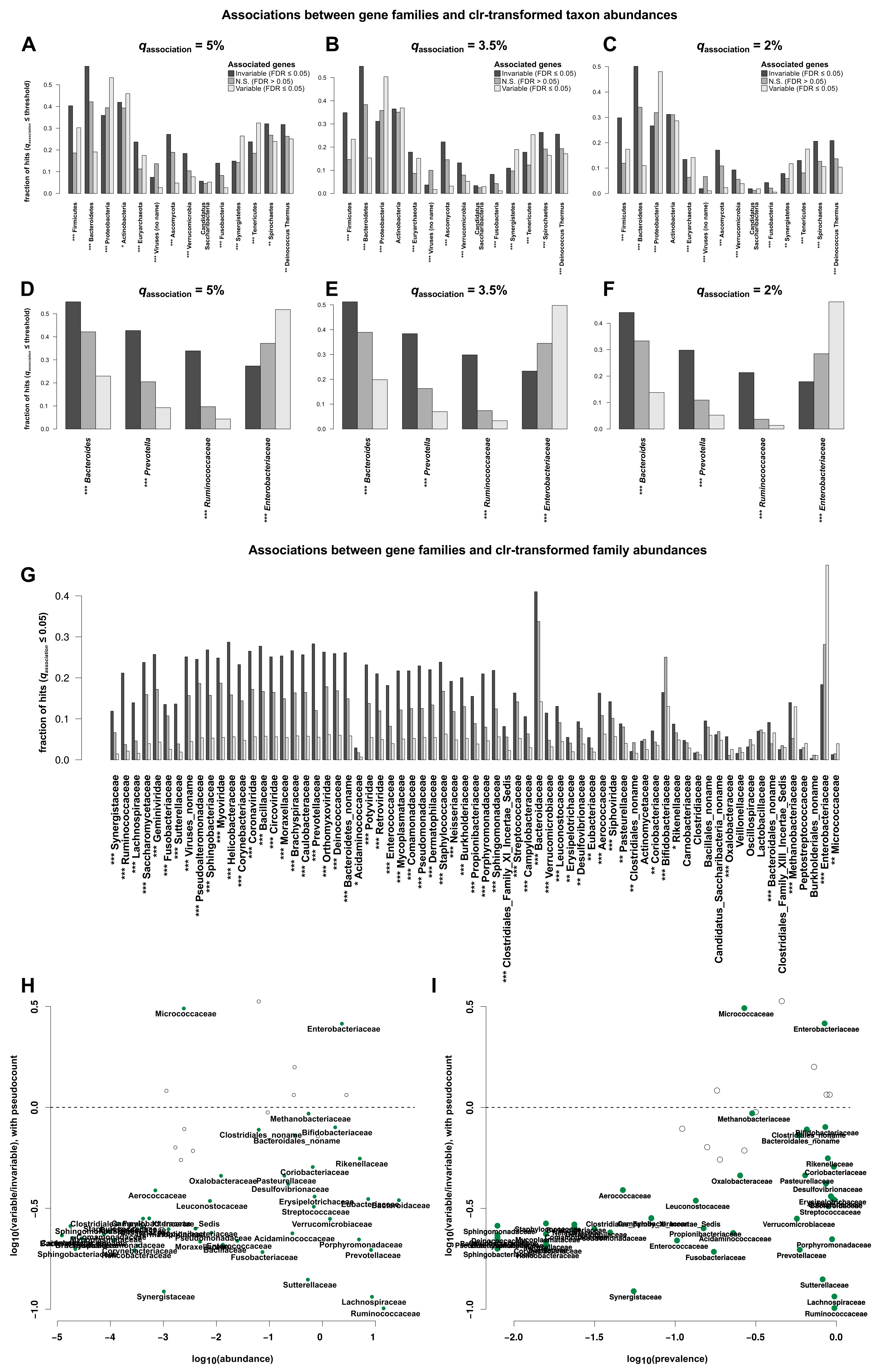

Associations between gene families and clr-transformed taxon abundances
A
$q_{association}$ = 5%
B
$q_{association}$ = 3.5%
C
$q_{association}$ = 2%
Associated genes
Invariable (FDR ≤ 0.05)
N.S. (FDR > 0.05)
Variable (FDR ≤ 0.05)
fraction of hits ($q_{association}$ ≤ threshold)
*** Firmicutes
*** Bacteroidetes
*** Proteobacteria
* Actinobacteria
*** Euryarchaeota
*** Viruses (no name)
*** Ascomycota
*** Verrucomicrobia
Candidatus Saccharibacteria
*** Fusobacteria
*** Synergistetes
*** Tenericutes
** Spirochaetes
** Deinococcus Thermus
D
$q_{association}$ = 5%
E
$q_{association}$ = 3.5%
F
$q_{association}$ = 2%
*** Bacteroides
*** Prevotella
*** Ruminococcaceae
*** Enterobacteriaceae
Associations between gene families and clr-transformed family abundances
G
fraction of hits ($q_{association}$ ≤ 0.05)
*** Synergistaceae
*** Ruminococcaceae
*** Lachnospiraceae
*** Saccharomycetaceae
*** Geminiviridae
*** Fusobacteriaceae
*** Sutterellaceae
*** Viruses_noname
*** Pseudoalteromonadaceae
*** Sphingobacteriaceae
*** Myoviridae
*** Helicobacteraceae
*** Corynebacteriaceae
*** Coronaviridae
*** Bacillaceae
*** Circoviridae
*** Moraxellaceae
*** Brachyspiraceae
*** Caulobacteraceae
*** Prevotellaceae
*** Orthomyxoviridae
*** Deinococcaceae
*** Bacteroidetes_noname
* Acidaminococcaceae
*** Potyviridae
*** Retroviridae
*** Enterococcaceae
*** Mycoplasmataceae
*** Comamonadaceae
*** Pseudomonadaceae
*** Dermatophilaceae
*** Staphylococcaceae
*** Neisseriaceae
*** Burkholderiaceae
*** Propionibacteriaceae
*** Porphyromonadaceae
*** Sphingomonadaceae
*** Clostridiales_Family_XI_Incertae_Sedis
*** Streptococcaceae
*** Campylobacteraceae
*** Bacteroidaceae
*** Verrucomicrobiaceae
*** Leuconostocaceae
** Erysipelotrichaceae
** Desulfovibrionaceae
** Eubacteriaceae
*** Aerococcaceae
*** Siphoviridae
** Pasteurellaceae
** Clostridiales_noname
Actinomycetaceae
** Coriobacteriaceae
*** Bifidobacteriaceae
* Rikenellaceae
Carnobacteriaceae
Clostridiaceae
Bacillales_noname
Candidatus_Saccharibacteria_noname
*** Oxalobacteraceae
Veillonellaceae
Oscillospiraceae
Lactobacillaceae
*** Bacteroidales_noname
Clostridiales_Family_XIII_Incertae_Sedis
*** Methanobacteriaceae
Peptostreptococcaceae
Burkholderiales_noname
*** Enterobacteriaceae
** Micrococcaceae
H
log10(variable/invariable), with pseudocount
log10(abundance)
I
log10(prevalence)
Micrococcaceae
Enterobacteriaceae
Methanobacteriaceae
Clostridiales_noname
Bifidobacteriaceae
Bacteroidales_noname
Rikenellaceae
Coriobacteriaceae
Oxalobacteraceae
Pasteurellaceae
Desulfovibrionaceae
Aerococcaceae
Leuconostocaceae
Erysipelotrichaceae
Eubacteriaceae
Bacteroidaceae
Streptococcaceae
Verrucomicrobiaceae
Acidaminococcaceae
Porphyromonadaceae
Prevotellaceae
Enterococcaceae
Fusobacteriaceae
Propionibacteriaceae
Moraxellaceae
Bacillaceae
Pseudomonadaceae
Comamonadaceae
Corynebacteriaceae
Helicobacteraceae
Sphingomonadaceae
Sphingobacteriaceae
Sutterellaceae
Synergistaceae
Lachnospiraceae
Ruminococcaceae
